# An AMP‐activated protein kinase‐PGC‐1α axis mediates metabolic plasticity in glioblastoma

**DOI:** 10.1002/ctm2.70030

**Published:** 2024-11-17

**Authors:** Benedikt Sauer, Jan Kueckelhaus, Nadja I. Lorenz, Süleyman Bozkurt, Dorothea Schulte, Jan‐Béla Weinem, Mohaned Benzarti, Johannes Meiser, Hans Urban, Giulia Villa, Patrick N. Harter, Christian Münch, Johannes Rieger, Joachim P. Steinbach, Dieter Henrik Heiland, Michael W. Ronellenfitsch

**Affiliations:** ^1^ Dr. Senckenberg Institute of Neurooncology University Hospital Goethe University Frankfurt Frankfurt am Main Germany; ^2^ University Cancer Center Frankfurt (UCT), University Hospital Goethe University Frankfurt Frankfurt am Main Germany; ^3^ German Cancer Consortium (DKTK), partner site Frankfurt, a partnership between DKFZ and University Hospital Frankfurt Goethe University Frankfurt Frankfurt am Main Germany; ^4^ Frankfurt Cancer Institute (FCI) University Hospital Goethe University Frankfurt Frankfurt am Main Germany; ^5^ Microenvironment and Immunology Research Laboratory Medical Center University of Freiburg Freiburg Germany; ^6^ Department of Neurosurgery Medical Center University of Freiburg Freiburg Germany; ^7^ Faculty of Medicine University of Freiburg Freiburg Germany; ^8^ Faculty of Medicine and Medical Center Comprehensive Cancer Center Freiburg (CCCF) University of Freiburg Freiburg Germany; ^9^ German Cancer Consortium (DKTK), partner site Freiburg, a partnership between DKFZ and University Medical Center Freiburg Freiburg Germany; ^10^ Institute of Molecular Systems Medicine, Faculty of Medicine Goethe University Frankfurt Frankfurt am Main Germany; ^11^ Institute of Neurology (Edinger Institute) University Hospital, Goethe University Frankfurt Frankfurt Germany; ^12^ Cancer Metabolism Group Department of Cancer Research Luxembourg Institute of Health Luxembourg Luxembourg; ^13^ Faculty of Science Technology and Medicine University of Luxembourg, 2 avenue de Université Esch‐sur‐Alzette Luxembourg; ^14^ German Cancer Consortium (DKTK), partner site Munich, a partnership between DKFZ and Ludwig‐Maximilians‐Universität (LMU) Munich Germany; ^15^ Center for Neuropathology and Prion Research, Faculty of Medicine Ludwig‐Maximilians‐Universität (LMU) Munich Germany; ^16^ Cardio‐Pulmonary Institute Goethe University Frankfurt Frankfurt am Main Germany; ^17^ Division of Neuro‐Oncology Hertie Institute of Clinical Brain Research University Hospital Tübingen Tübingen Germany

**Keywords:** AMP‐activated protein kinase, glioblastoma, hypoxia, metabolic plasticity, PGC‐1α, PPARGC1A, tumour microenvironment

## Abstract

Glioblastoma, the most frequent primary malignant brain tumour in adults, is characterised by profound yet dynamic hypoxia and nutrient depletion. To sustain survival and proliferation, tumour cells are compelled to acquire metabolic plasticity with the induction of adaptive metabolic programs. Here, we interrogated the pathways necessary to enable processing of nutrients other than glucose.

We employed genetic approaches (stable/inducible overexpression, CRISPR/Cas9 knockout), pharmacological interventions with a novel inhibitor of AMP‐activated protein kinase (AMPK) in glioblastoma cell culture systems and a proteomic approach to investigate mechanisms of metabolic plasticity. Moreover, a spatially resolved multiomic analysis was employed to correlate the gene expression pattern of PGC‐1α with the local metabolic and genetic architecture in human glioblastoma tissue sections.

A switch from glucose to alternative nutrients triggered an activation of AMPK, which in turn activated PGC‐1α‐dependent adaptive programs promoting mitochondrial metabolism. This sensor‐effector mechanism was essential for metabolic plasticity with both functional AMPK and PGC‐1α necessary for survival and growth of cells under nonglucose nutrient sources. In human glioblastoma tissue specimens, PGC‐1α‐expression correlated with nonhypoxic tumour niches defining a specific metabolic compartment.

Our findings reveal a cell‐intrinsic nutrient sensing and switching mechanism. The exposure to alternative fuels triggers a starvation signal that subsequently is passed on via AMPK and PGC‐1α to induce adaptive programs necessary for broader spectrum nutrient metabolism. The integration of spatially resolved transcriptomic data confirms the relevance of PGC‐1α especially in nonhypoxic tumour regions. Thus, the AMPK‐PGC‐1α axis is a candidate for therapeutic inhibition in glioblastoma.

**Key Points/Highlights:**

- AMPK activation induces PGC‐1α expression in glioblastoma during nutrient scarcity.
- PGC‐1α enables metabolic plasticity by facilitating metabolism of alternative nutrients in glioblastoma.
- PGC‐1α expression is inversely correlated with hypoxic tumour regions in human glioblastomas.

## Graphical abstract

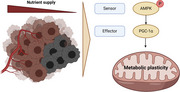

## 1 INTRODUCTION

In adults, glioblastoma (GB) is the primary malignant brain tumour with the highest incidence. Despite multimodal treatment this tumour entity still carries a dismal prognosis.^1^ A characteristic feature of GB is its highly heterogeneous microenvironment with oxygen tensions ranging from 0.1% to 10%.^2^ To sustain growth under such conditions, tumour cells must activate metabolic adaptive programs. Historically, GBs were assumed to meet their energy demand mainly via glycolysis disregarding oxidative metabolism, even when oxygen is available (i.e. aerobic glycolysis). However, this characteristic trait known as the Warburg effect^3^ has been challenged in recent years. In this respect, GB cells have been shown to be able to metabolise nutrient sources other than glucose for energy production and are able to maintain oxidative phosphorylation down to oxygen tensions of around 1%.^4^ Fatty acid oxidation in particular was identified as a metabolic pathway whose inhibition resulted in slower tumour growth and thus a prolongation of survival in mouse models.^5^, ^6^ These findings, contrasting Warburg's original hypothesis, emphasise the importance of oxidative metabolism and the utilisation of a broader spectrum of nutrient sources in GB. While glycolysis enables cells to produce at least some energy from glucose bypassing mitochondria, most alternative nutrient sources require oxidative phosphorylation. From a cellular perspective, declining energy storages should be the signal that recuperation is necessary and alternative nutrient sources must be tapped. While this is certainly a clonal advantage, the mechanisms of how metabolic plasticity can be achieved in GB cells have, to our knowledge, not been understood in detail.

The heterotrimeric protein AMP‐activated protein kinase (AMPK) consists of one each of several isoforms of α, β and γ subunits. Activation is conferred by increasing concentrations of AMP that inversely correlate with the cellular energy load.^7^ AMPK‐responsive metabolic pathways have a critical impact on energy metabolism and promote adenosine triphosphate (ATP)‐generating catabolism while simultaneously diminishing biosynthetic pathways that would consume ATP during energy deficiency.^7^, ^8^ A major target of AMPK is the peroxisome proliferator‐activated receptor gamma coactivator 1‐alpha (PGC‐1α) (encoded by the gene *PPARGC1A*).^9^, ^10^ PGC‐1α promotes mitochondrial metabolism and cellular respiration and is therefore also referred to as the ‘master regulator’ of mitochondrial biogenesis.^11^ We have previously shown that PGC‐1α is a key factor for the maintenance of the neoplastic phenotype of glioblastoma cells^12^ in line with findings of other groups that PGC‐1α is crucial for motility and metastasis in breast cancer.^13^ Recently, a transcriptomic‐based analysis of human GBs enabled the definition of several pathway‐based subtypes.^14^ One specific subtype, coined the mitochondrial subtype (MTC), displayed enhanced mitochondrial metabolism and was sensitive towards silencing of PGC‐1α which was also identified as a subtype‐specific master regulator for MTC‐GBs.^14^ Following technical advances more complex spatially resolved multiomics approaches including transcriptomics, metabolomics, and proteomics have been utilised to define regional transcriptional and metabolic GB signatures that highlight spatial heterogeneity between several segregated niches.^15^

In our study, we describe a novel mechanism of metabolic plasticity with AMPK as the energy sensor and PGC‐1α as the effector that enables GB cells to utilise alternative fuels. We report that the expression of PGC‐1α is closely linked to the local tumour microenvironment in human GB tissue samples and is specifically found in nonhypoxic tumour niches. Proteomic analyses further indicate that PGC‐1α expression is linked to a profile of oxidative metabolism. Translating our results to nontransformed and other cancer cells, we show that this mechanism could be a conserved biological process of cell intrinsic metabolic plasticity. From a therapeutic perspective, targeting mechanisms of metabolic plasticity could be a promising approach in the treatment of solid tumours.

## 2 MATERIALS AND METHODS

### 2.1 Cell lines, reagents and culture conditions

Reagents, cell lines and culture conditions are specified in the Supplementary Materials and Methods.

### 2.2 Stable and inducible overexpression

Stable transfection of PGC‐1α was performed using the vector pcDNA3.1 PGC‐1α (clone ID: OHu27412D) purchased from GenScript (Piscataway, NJ, USA). As a vector control, the sequence was deleted to generate the ‘empty’ pcDNA3.1 plasmid. After transfection of the cells with Attractene transfection reagent (Qiagen, Hilden, Germany), selection with G418 (neomycin) (400 µg/mL) was performed. For inducible PGC‐1α overexpression, the pTetOne system (Takara Clontech #634301; Saint‐Germain‐en‐Laye, France) was used. Using NotI and MluI cloning sites, the PGC‐1α sequence was recloned (Genscript, Piscataway Township, NJ, USA). Transfection of the pTetOne plasmid including a linear puromycin marker was performed using Xfect transfection reagent according to the manufacturer's protocol, followed by selection and cultivation of cells in medium containing puromycin (2 µg/mL).

### 2.3 CRISPR/Cas9 knockout cell generation

To generate the CRISPR/Cas9 knockout of AMPK α1 and 2 subunits target plasmids for AMPK α1 and α2 (pX462‐hPRKAA1‐gRNA, pX462‐hPRKAA2‐gRNA, #74374 and #74377) were purchased from Addgene (Watertown, MA, USA). AMPK knockout cells have been described previously.^16^ AMPK expression of each cell clone was analysed by immunoblot and the most suitable clones were selected for the experiments.

### 2.4 Generation of Rho0 cells

Rho0 cells were generated as described previously.^17^ LNT‐229 cells were cultured for > 2 months in DMEM supplemented with (LNT‐229 Rho0 cells) or not supplemented with (LNT‐229 Rho+ cells) ethidium bromide (200 ng/mL). Uridine (50 µg/mL) was complemented to the culture medium.

### 2.5 Induction of hypoxia

Induction of hypoxia was carried out as reported previously.^18^, ^19^ After seeding, cells were left to attach in full medium overnight in normoxia. On the following day, the medium was exchanged and the cells were cultured with the specific experimental condition. To achieve 0.1% oxygen the cells were incubated in Gas Pak pouches as described previously (Becton‐Dickinson, Heidelberg, Germany).^18^ 1% oxygen was induced by a Labotect incubator (Göttingen, Germany).^19^

### 2.6 Extraction of RNA and quantitative reverse transcription‐PCR (qRT‐PCR) analysis

The qPCR protocol has been described previously.^19^ For RNA isolation the TRIzol® as well as the RNAeasy Kit (Invitrogen, Karlsruhe, Germany) were used. CDNA was created using the Vilo cDNA synthesis kit (Invitrogen). QPCR was conducted with the IQ5 RT‐PCR detection system (Biorad, Munich, Germany) using Absolute Blue Q‐PCR Mastermix with Sybr Green+Fluorescein (Thermo Fisher Scientific, Hamburg, Germany). For normalisation, *18S* and *SDHA* were used as housekeeping genes. Normalisation of cycle threshold (Ct) values was performed relative to the amplification of *18S* and *SDHA*.^20^ Primer pair sequences are included in the supplement (Table S2).

### 2.7 Lysate preparation and immunoblot analysis

Cell lysates were prepared following a standard protocol. After dilution in Laemmli buffer, proteins were separated by size with SDS‐PAGE. Subsequently, protein samples were transferred to a nitrocellulose membrane with a pore size of 0.45 µm (GE Healthcare, Little Chalfont, UK) with a ‘wet blotting’ technique.^21^ Incubation with the respective primary antibodies was done overnight. As a next step, membranes were incubated with the according secondary antibody (Table S3). For band detection, we used a solution mix containing 1 mL solution A (200 mL 0.1 M Tris‐HCl pH 8.6, 50 mg luminol), 100 µL solution B (11 mg p‐hydroxycoumaric acid, 10 mL DMSO), and 0.3 µL H_2_O_2_ (30%).

### 2.8 Determination of cell density and viability

Crystal violet staining was used for the determination of cell density.^22^, ^23^ Before experiments, all wells were checked for cellular aggregates that could impair data quality. Cell viability was assessed by quantifying propidium iodide (PI) uptake using flow cytometry on aBD Canto II flow cytometer. Data analysis was performed with the Diva software.^24^ Also, cell viability was determined by quantifying lactate dehydrogenase (LDH) release using the LDH cytotoxicity assay kit (Roche, Basel, Switzerland).^21^ Prior to the start of viability analyses, matching cell densities were ensured by a parallel experiment using crystal violet staining.

### 2.9 Measurement of glucose and galactose

After incubation with the respective experimental conditions, a cell‐free supernatant was obtained by centrifugation (300 *g*, 5 min, 4°C). Subsequently, concentrations of glucose and lactate were accessed by the Hitachi 917 biochemical analyser (Central Laboratory of the University Hospital Frankfurt). For determination of galactose concentration, the Galactose Assay Kit (Sigma Aldrich, Darmstadt, Germany) was used according to the manufacturer's protocol. Absorbance at 570 nm was determined using the SPARK® Multimode Microplate Reader (Tecan, Männedorf, Switzerland).

### 2.10 Oxygen consumption

Prior to the start of analyses, matching cell numbers were ensured by a parallel experiment using crystal violet staining.^24^ Measurement of oxygen consumption was performed as described previously.^21^ In short, glioma cells were covered with a layer of paraffin oil and consumption of oxygen was assessed in specialised plates containing oxygen sensors at the well´s base (PreSens, Regensburg, Germany).^25^ Statistical analysis was performed as end point analysis.

### 2.11 Analysis of reactive oxygen species

For detection of cytosolic ROS, cells were kept for 10 h in glutamine‐free medium containing 2 mM glucose without serum. Subsequently, the cells were washed with 1× PBS followed by an incubation step with 10 µM of the fluorescent dye dichlorodihydrofluorescein diacetate (H2DCFDA) for 30 min at 37°C. Intensity of fluorescence was then determined by flow cytometry (FACS) using a BD Canto II (BD Biosciences, San Jose, CA, USA). Data analysis was performed with the corresponding Diva software.

### 2.12 Cellular ATP measurement

Assessment of intracellular ATP levels was carried out as described earlier.^18^ In short, ATP releasing agent (Sigma‐Aldrich; Merck KGaA) was used for lysis of the cells. Measurement of ATP concentration was performed using the luciferase‐based CLS II kit (Boehringer) according to the manufacturers protocol.

### 2.13 Chorio‐allantoic membrane (CAM) assay

Chicken eggs (breed: Leghorn, LSL‐Rhein‐Main Geflügelvermehrungsbetriebe GmbH) were purchased 3 days after fertilisation and kept for 7 days at 37°C, 5% CO_2_ as well as high humidity and turned manually for 180° every 48 h. On day 8, the air chamber of the eggs was opened and the underlying CAM was lightly stimulated with a cotton swab. A 5.00 × 1.50 mm rubber sealing ring was placed on a large calibre vessel running inside the CAM. 1 × 10^6^ cells, dissolved in 20 µL of an equal ratio mixture of Matrigel (Corning, New York, USA) and DMEM, were added into the ring. The eggs were sealed airtight with clear scotch tape and incubated for another 7 days as described above. Subsequently the tumour was separated from the CAM and fixed in 4% paraformaldehyde (PFA). The weight of the tumours was determined using a precision balance.

### 2.14 Mass spectrometry

Cells were seeded in triplicates for proteomic analysis. Sample preparation and proteome analysis followed previously established methods.^26^ Additional details regarding sample preparation, fractionation and liquid chromatography mass spectrometry have been included in the Supplementary Materials and Methods.

### 2.15 Preprocessing

MassSpec data were loaded into R software (version 4.2.2) and normalised using quantile normalisation. Differential expression analysis between the empty vector and the PGC‐1α overexpression cells was performed by the Wilcoxon Rank Sum test. The top 20 differentially expressed proteins were visualised in a heatmap using the heatmap package.

### 2.16 Protein network analysis

Protein co‐correlation networks analysis was performed by WGCNA (weighted correlation network analysis) including the cell line and experimental condition as trait features. First, we calculated the optimal soft‐power to achieve scale‐free topology (SFT) by fitting an SFT model with various soft‐power thresholds *p* = {1,…,20}, determining that 16 was the optimal value. Next, we constructed a signed co‐expression network using the topological overlap matrix (TOM) via the hdWGCNA::ConstructNetwork function. The co‐expression network was visualised using a Uniform Manifold Approximation and Projection for dimension reduction (ModuleUMAPPlot). Next, hub genes of each module were quantified by computing module connectivity (ModuleConnectivity) followed by a gene ontology analysis using the top 100 module associated genes (compareCluster). Visualisation of pathways related to the identified modules, was achieved with the clusterProfiler package (dotplot) and the correlation between each co‐expression module to the condition and cell line status were computed.

### 2.17 Single cell analysis

To provide context for the protein modules strongly linked to overexpression of PGC‐1α, we summarised the hub genes and evaluated their expression in the GBMap single cell reference dataset. This dataset was obtained and processed using the Seurat package, with hub genes identified through Seurat's addFeature function. The visualisation was performed by projecting the model expression into the cell UMAP provided by the GBMap integration algorithm including over 1 million cells.

### 2.18 Spatial multiomic analysis

To conduct integrative analysis, we utilised a recently released multiomics dataset,^15^ integrating Matrix Assisted Laser Desorption/Ionisation (MALDI) and array‐based spatial transcriptomics (Visium 10X technology). The downstream analysis and visualisation were carried out using the SPATA framework via the SPATA2 package (available at https://github.com/theMILO lab/SPATA2) and the SPATAwrappers (accessible at https://github.com/heilandd/SPATAwrappers). Surface plots overlaid with H&E images were generated using SPATA2's plotSurface function, where genes were colour‐coded and smoothed, and transparency was adjusted using gene‐specific parameters. Spatially weighted correlation analysis was conducted using SPATAwrappers' runSpatialRegression function with the model = ‘CCA’ configuration, comparing metabolic and transcriptomic features. In the MALDI dataset, target metabolites were identified using the METASPACE database (available at https://metaspace2020.eu).

### 2.19 Statistical analysis

The quantitative data are reported with the indicated standard deviation (S.D.). *p* Values were derived from two‐tailed student's *t*‐tests (Excel, Microsoft, Seattle, WA, USA). *p* Values < 0.01 and < 0.05 were considered highly significant (**) and significant (*). Values of *p* > 0.05 were considered not significant (n.s.).

## 3 RESULTS

### 3.1 Glioblastoma cells are competent to adapt to alternative nutrient sources

**FIGURE 1:**
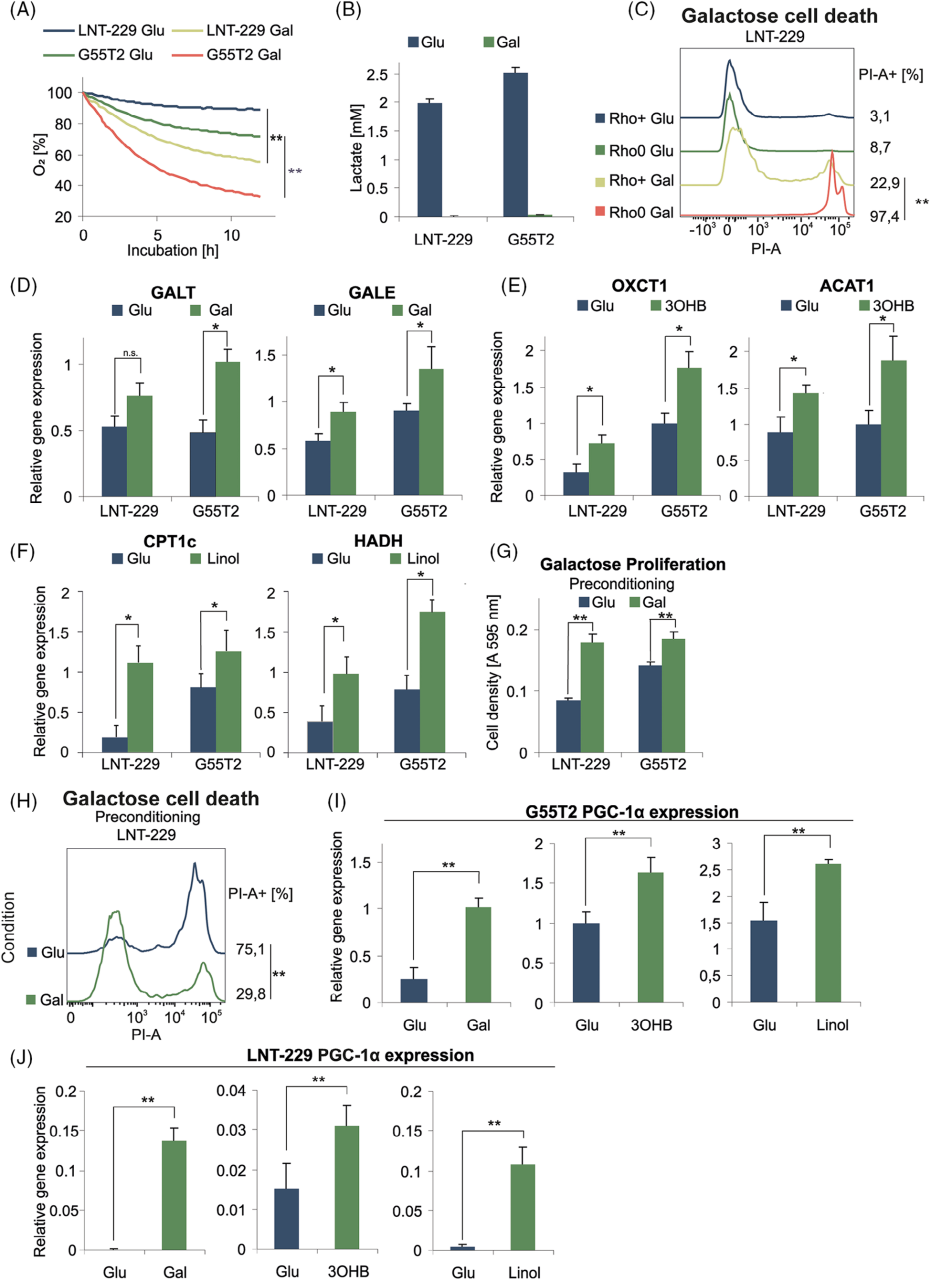
Glioma cells are able to adapt to different nutrients. (A) LNT‐229 and G55T2 cells were incubated for 12 h in serum‐free medium without glutamine containing either 25 mM glucose (Glu) or galactose (Gal). Oxygen consumption was measured by a fluorescence‐based assay (*n* = 3, mean, **p < 0.01). (B) Cells were incubated for 8 h in serum‐free medium without glutamine containing either 25 mM glucose or galactose. Lactate production was quantified in the supernatant (*n* = 3, mean ± SD). (C) LNT‐229 Rho+ and Rho0 cells were incubated in serum‐free medium without glutamine containing either 25 mM glucose or galactose. Cell death was determined by propidium iodide staining after 24 h. The percentage of propidium iodide‐positive cells (PI‐A +) is indicated. (D–F) cDNA of LNT‐229 and G55T2 cells cultured for 5 days in full medium without glutamine and either 25 mM glucose or 25 mM galactose (D) and in serum‐free medium without glutamine containing 2 mM glucose with or without the addition of 5 mM hydroxybutyrate (3OHB) (E) or 100 µM linoleic acid (Linol) (F) for 5 days was generated. Gene expression of GALT and GALE (D), OXCT1 and ACAT1 (E), and CPT1c and HAHD (F) was quantified (*n* = 3, mean ± SD, *p < 0.05, **p < 0.01). (G, H) LNT‐229 and G55T2 cells were preconditioned in culture medium containing 25 mM glucose or 25 mM galactose for 5 days and were then incubated in serum‐free medium without glutamine containing 25 mM glucose or 25 mM galactose. Cell density was measured by crystal violet staining after 3 days (G); cell death was quantified by propidium iodide staining. The percentage of propidium iodide positive cells (PI‐A +) is indicated (*n* = 3, representative curves are displayed, **p < 0.01) (H). (I, J) Gene expression of PGC‐1α was quantified by qPCR using the cDNA described in D–F.

We hypothesised that tumour cells can be conditioned to alternative nutrient sources by replacing glucose with galactose, ketone bodies or fatty acids. Galactose as a nutrient was chosen to induce cellular oxidative phosphorylation.^27^, ^28^, ^29^, ^30^ The conversion of galactose to enter glycolysis is restricted by slow enzyme kinetics, which is why anaerobic galactose metabolism would not suffice to cover the cellular energy demand mandating additional mitochondrial ATP generation.^31^, ^32^ We measured oxygen consumption under glucose and galactose in LNT‐229 und G55T2 GB cells that had previously been cultured under standard conditions with abundant glucose. As expected, incubation with galactose triggered enhanced consumption of oxygen (Figure 1A). No lactate production was detectable indicating that the glycolytic end product pyruvate was channelled further into the citric acid cycle to undergo oxidative phosphorylation (Figure 1B). In line, incubation with galactose led to an increased rate of cell death in mitochondria‐depleted Rho0 cells^33^ (Figure 1C), as well as in hypoxia (Figure S1A). Exposure to galactose caused some cell death in glioma cells compared to glucose already in normoxia (Figure 1C). Gene expression of enzymes of galactose metabolism including galactose‐1‐phosphate uridyltransferase (GALT) and UDP‐glucose 4‐epimerase (GALE) was induced in G55T2 cells when glucose was substituted with galactose in the culture medium. In LNT‐229 cells, only GALE‐expression was significantly increased (Figure 1D). In a similar manner, key enzymes of ketone body (ACAT1 and OXCT1) and fatty acid metabolism (CPT1c and HADH) displayed elevated gene expression levels when hydroxybutyrate (3OHB) or linoleic acid were added under glucose restricted conditions (Figure 1E and F). When the same experiment was performed under glucose abundance, the addition of alternative nutrients had no effect on gene expression levels of the above‐mentioned enzymes (data not shown). Long‐term cultivation of LNT‐229 and G55T2 cells was possible in full medium with galactose (replacing glucose). We hypothesised that the cells would get attuned and develop a growth advantage in galactose (compared to cells preconditioned in glucose containing medium). Indeed, galactose preconditioned cells displayed a higher cell density and lower amount of cell death in galactose medium (Figure 1G and H). This indicates that metabolic adaptation must have occurred. Because mitochondria are mandatory for the utilisation of the alternative nutrients, we hypothesised that PGC‐1α as a master regulator of mitochondrial metabolism was involved. Indeed, when incubated with linoleic acid, 3OHB or galactose, LNT‐229 and G55T2 cells increased their PGC‐1α expression levels (Figure 1I and J).

### 3.2 PGC‐1α induces oxygen consumption and confers a growth advantage in a CAM tumour model

**FIGURE 2:**
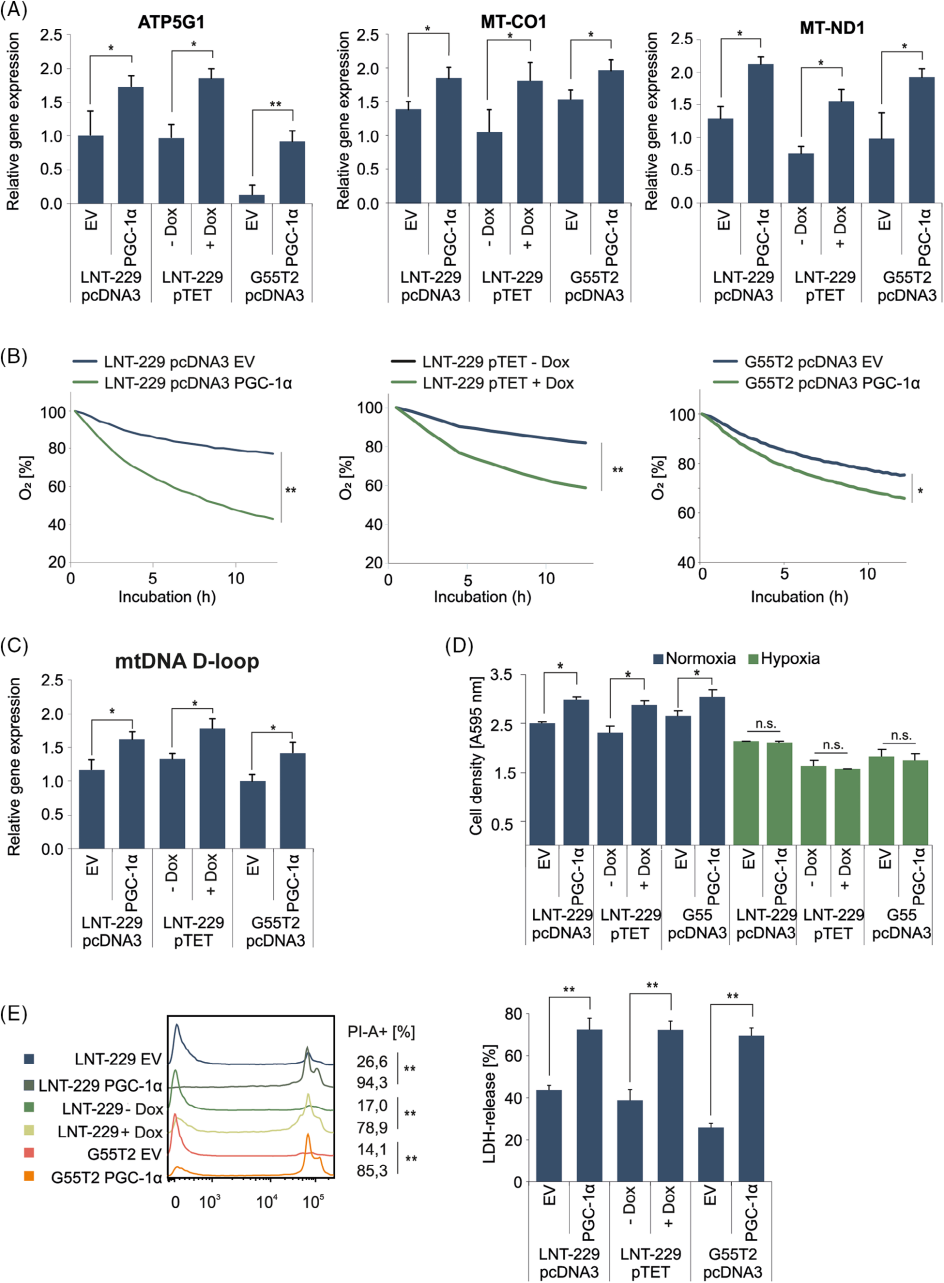
Overexpression of PGC‐1α induces an oxidative phenotype. (A) cDNA of empty vector (EV) and PGC‐1α overexpressing cells cultured in standard conditions was generated. Gene expression of ATP5G1, MT‐CO1 and MT‐ND1 was quantified by qPCR (*n* = 3, mean ± SD, *p < 0.05, **p < 0.01). (B) Cells were incubated in medium containing 10% FCS with 25 mM glucose. Oxygen consumption was measured by a fluorescence‐based assay (*n* = 3, mean, **p < 0.01). (C) cDNA of control and PGC‐1α overexpressing cells generated in (A) was analysed for expression of mtDNA D‐loop by qPCR (*n* = 3, mean ± SD, *p < 0.05). (D) Cells were incubated in culture‐medium containing 25 mM glucose and 10% FCS under normoxic conditions and 1% oxygen. Cell density was measured by crystal violet staining after 3 days (*n* = 3, mean ± SD). (E) Cells were exposed to glucose restricted (2 mM glucose) serum‐free DMEM under normoxic conditions (data not shown) or 0.1% oxygen until cell death (28 h). Cell death was quantified by by propidium iodide staining (left panel) and LDH release (right panel). The percentage of propidium iodide positive cells (PI‐A +) is indicated (*n* = 3, representative curves are displayed, **p < 0.01) as well as LDH‐release (*n* = 4, mean ± SD, **p < 0.01).

To gain further insight into the effects mediated by PGC‐1α, we investigated PGC‐1α overexpressing cells. We have previously shown that PGC‐1α expression differs between established glioma cell lines.^12^ We selected two cell lines (LNT‐229 and G55T2) with low PGC‐1α expression for overexpression. QPCR confirmed stable overexpression in LNT‐229 and G55T2 cells and doxycycline inducible overexpression in LNT‐229 cells after transfection (Figure S1B). Immunoblot analysis verified elevated protein levels of PGC‐1α in PGC‐1α overexpressing cells compared to control cells (Figure S1C). We hypothesised that overexpression of PGC‐1α would induce a switch towards a more oxidative metabolic phenotype. Consistent with this, quantification of gene expression of the c1 subunit of mitochondrial ATP synthase (*ATP5G1*), *MT‐CO1*, and subunit 1 of NADH dehydrogenase (*MT‐ND1*) revealed higher expression levels in PGC‐1α‐overexpressing cells (Figure 2A). Mitochondrially coded Cytochrome b (*MT‐CYB*) displayed higher expression levels only in LNT‐229 cells (Figure S1D). To analyse functional effects, we measured oxygen consumption which was enhanced in PGC‐1α overexpressing cells (Figure 2B), indicating an elevated rate of oxidative phosphorylation. In line with this, PGC‐1α overexpressing cells also displayed a higher mitochondrial content as indicated by enhanced expression of the mitochondrial (mt) DNA D‐loop (Figure 2C). Furthermore, we observed elevated levels of reactive oxygen species (ROS) in PGC‐1α overexpressing cells compared to the empty vector control cells in line with the increased rate of cellular respiration because ROS occur as a by‐product of the respiratory chain (Figure S1E). At the same time, gene expression of the ROS detoxifying enzymes SOD1 and SOD2, known PGC‐1α targets, was elevated in LNT‐229 PGC‐1α overexpressing cells, while no difference was detectable in LNT‐229 PGC‐1α overexpressing cells (Figure S1F). Glucose consumption remained unaltered between the corresponding cells (Figure S1G). In line with previous results, PGC‐1α overexpressing cells displayed increased intracellular ATP levels in comparison to the respective control cells (Figure S1H). Overexpression of PGC‐1α resulted in a higher cell density under normoxic conditions whereas under moderate hypoxia (1% O_2_) no difference was detectable (Figure 2D). We hypothesised that the enhanced rate of oxidative phosphorylation could be a liability in oxygen‐restricted scenarios. To this end, we conducted cell viability assays in severe hypoxia (0.1% O_2_) in which PGC‐1α overexpressing cells displayed increased susceptibility towards hypoxia‐induced cell death (Figure 2E). To test the growth of PGC‐1α‐overexpressing in an in vivo model, we performed a CAM assay. LNT‐229 empty vector, LNT‐229 PGC‐1α, and a 1:1 mixture of both subcell lines were analysed. After 7 days of incubation, the mixed cell population showed a trend towards the most prominent tumour growth, followed by the PGC‐1α overexpressing cells and the control cells, however, without achieving statistical significance (Figure S2A–D).

### 3.3 PGC‐1α overexpression facilitates metabolism of alternative nutrients

**FIGURE 3:**
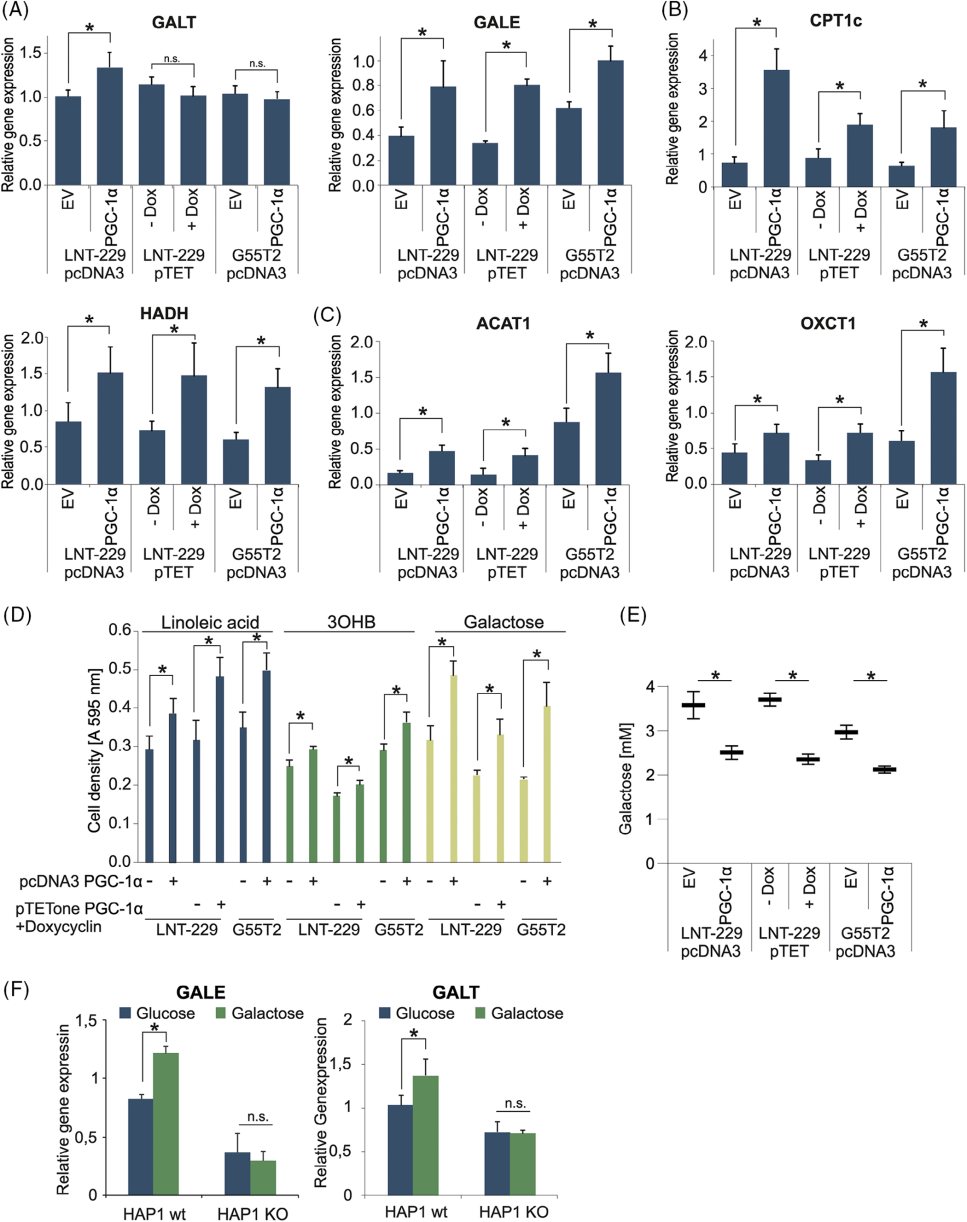
Overexpression of PGC‐1α facilitates use of alternative nutrients. (A–C) cDNA of LNT‐229 and G55T2 EV and PGC‐1α cells and LNT‐229 pTetOne PGC‐1α cells with and without 0.1 µg/mL doxycycline (± Dox) cultured in serum‐free medium for 24 h was generated. Gene expression of GALT and GALE (A), CPT1c and HAHD (B), and OXCT1 and ACAT1 (C) was quantified; values are normalised to 18S as well as SDHA housekeeping gene expression (*n* = 3, mean ± SD, *p < 0.05, **p < 0.01). (D) The cells were exposed to serum‐free medium without glutamine containing either 25 mM galactose or 2 mM glucose with the addition of 100 µM linoleic acid or 5 mM 3OHB. Cell density was measured by crystal violet staining after 3 days (*n* = 3, mean ± SD). (E) Cells were incubated in medium containing 5 mM galactose for 6 h. Remaining galactose in the medium was determined (n = 4, mean ± SD, *p < 0.05). (F) cDNA of HAP1 wt and HAP1 PGC‐1α KO cells cultured in 25 mM glucose or 25 mM galactose for 5 days was generated. Gene expression of GALE and GALT was quantified (n = 3, mean ± SD, *p < 0.05).

To test whether the adaptation to alternative nutrients (Figure 1E–K) could be attributed to PGC‐1α, we compared the corresponding gene expression profiles with those of PGC‐1α overexpressing cells. Indeed, expression of *GALE* (Figure 3A), *CPT1c* and *HAHD* (Figure 3B) and *OXCT1* and *ACAT1* (Figure 3C) was increased in all cell lines. *GALT* expression was only increased in LNT‐229 pcDNA3 PGC‐1α cells (Figure 3A). We presumed that this would benefit cells in conditions of low glucose, when alternative nutrients are present. In line with this notion, linoleic acid, 3OHB or galactose as nutrients caused a higher cell density in cells overexpressing PGC‐1α (Figure 3D). We concluded that PGC‐1α facilitated metabolism by ensuring sufficient mitochondrial capacity while simultaneously elevating the levels of the required enzymes. Exposing the cells to 1% hypoxia abrogated this effect, suggesting that sufficient oxygen is required for PGC‐1α to exert its effects (Figure S2E). To verify whether PGC‐1α enhances conversion of galactose, we determined galactose consumption. PGC‐1α overexpressing cells consumed significantly more galactose than control cells (Figure 3E). To further understand the relevance of PGC‐1α for metabolic plasticity, we analysed PGC‐1α deficient cells under galactose. While in wild‐type HAP1 cells gene expression levels of GALT and GALE were induced after a 5‐day incubation in culture medium with galactose (without glucose), PGC‐1α HAP1 knockout cells did not display any changes (Figure 3F). Because linoleic acid and 3OHB can only be metabolised via oxidative phosphorylation, we wondered whether their addition in glucose starved conditions would impact the cellular O_2_ consumption. We incubated LNT‐229 and G55T2 PGC‐1α overexpressing and control cells in low glucose medium with and without 3OHB. The addition of 3OHB led to elevated levels of oxygen consumption only in cells with overexpression of PGC‐1α (Figure S3A). In the same manner, we conducted the experiment with linoleic acid and additionally treated the cells with the fatty acid oxidation inhibitor etomoxir. Linoleic acid also resulted in an elevated oxygen consumption in PGC‐1α overexpressing cells. Etomoxir abrogated this effect and resulted in a generally lower oxygen consumption rate in both, PGC‐1α overexpressing and control cells (Figure S3B), which might be in part due to off‐target effects on mitochondrial respiratory chain complexes.^34^

### 3.4 PGC‐1α is enhanced following AMPK activation

**FIGURE 4:**
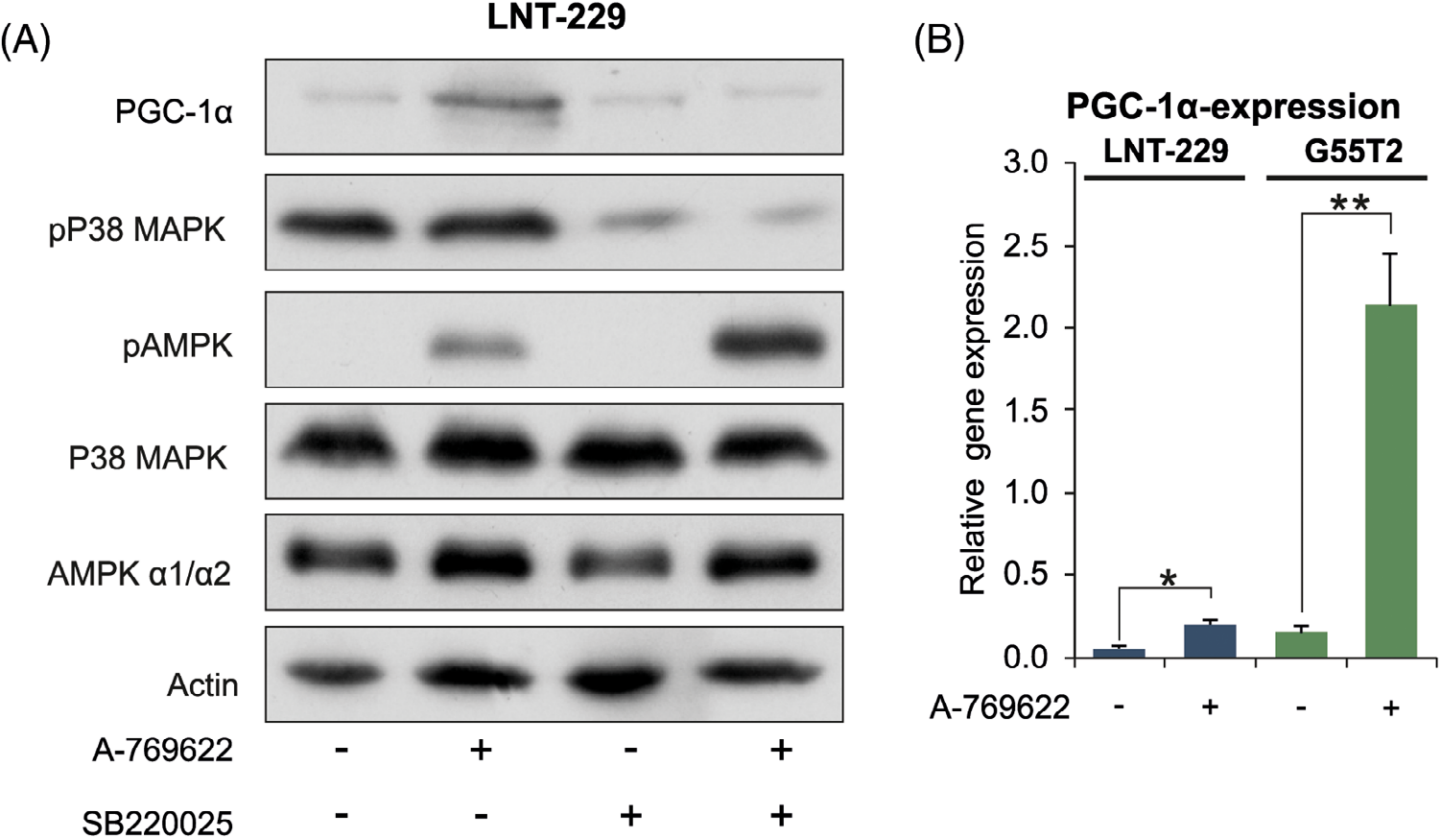
PGC‐1α is enhanced following AMPK activation. (A) LNT‐229 cells were incubated for 24 h in serum‐free medium and treated with 100 µM A‐769662 and 10 µM SB220025 as indicated. Cellular lysates were analysed by immunoblot with antibodies for PGC‐1α, Phospho‐p38 MAPK (Thr180/Tyr182), Phospho‐AMPKα (Thr172), p38 MAPK, AMPK α and actin. (B) cDNA of LNT‐229 and G55T2 cells treated with 100 µM A‐769662 for 24 h was generated. Gene expression of PGC‐1α was quantified; values are normalised to 18S as well as SDHA housekeeping gene expression (*n* = 3, mean ± SD, *p < 0.05, **p < 0.01).

For a mechanistic analysis of PGC‐1α regulation during metabolic adaption to nonglucose nutrients (Figure 1I and J), we examined the AMPK‐PGC‐1α axis. It has previously been shown that the AMP‐activated protein kinase (AMPK) is a regulator of PGC‐1α in cancer cells via the p38 mitogen‐activated protein kinase (p38 MAPK).^9^ Indeed, in glioma cell lines, pharmacological AMPK activation with the compound A‐769662 increased mRNA and protein levels of PGC‐1α, which could be abrogated by addition of the p38 MAPK inhibitor SB220025 (Figure 4A and B). To test if AMPK was active in our experimental conditions, we incubated LNT‐229 and G55T2 cells in medium containing low and high concentrations of glucose or galactose. Exposure to galactose or low glucose concentration with simultaneous supplementation of linoleic acid or 3OHB increased AMPK activity indicated by elevated phosphorylation levels of the α‐subunit and its downstream target acetyl‐CoA carboxylase (ACC) (Figure S3C). Interestingly, in this experiment PGC‐1α‐levels were only elevated in LNT‐229 cells incubated with galactose, in G55T2 cells there was only a marginal trend, indicating a temporal delay between AMPK activation and increase in PGC‐1α protein expression that is better detectable after a longer incubation period (Figure S4B).

### 3.5 Knockout of AMPK catalytic subunits disrupts galactose metabolism

**FIGURE 5:**
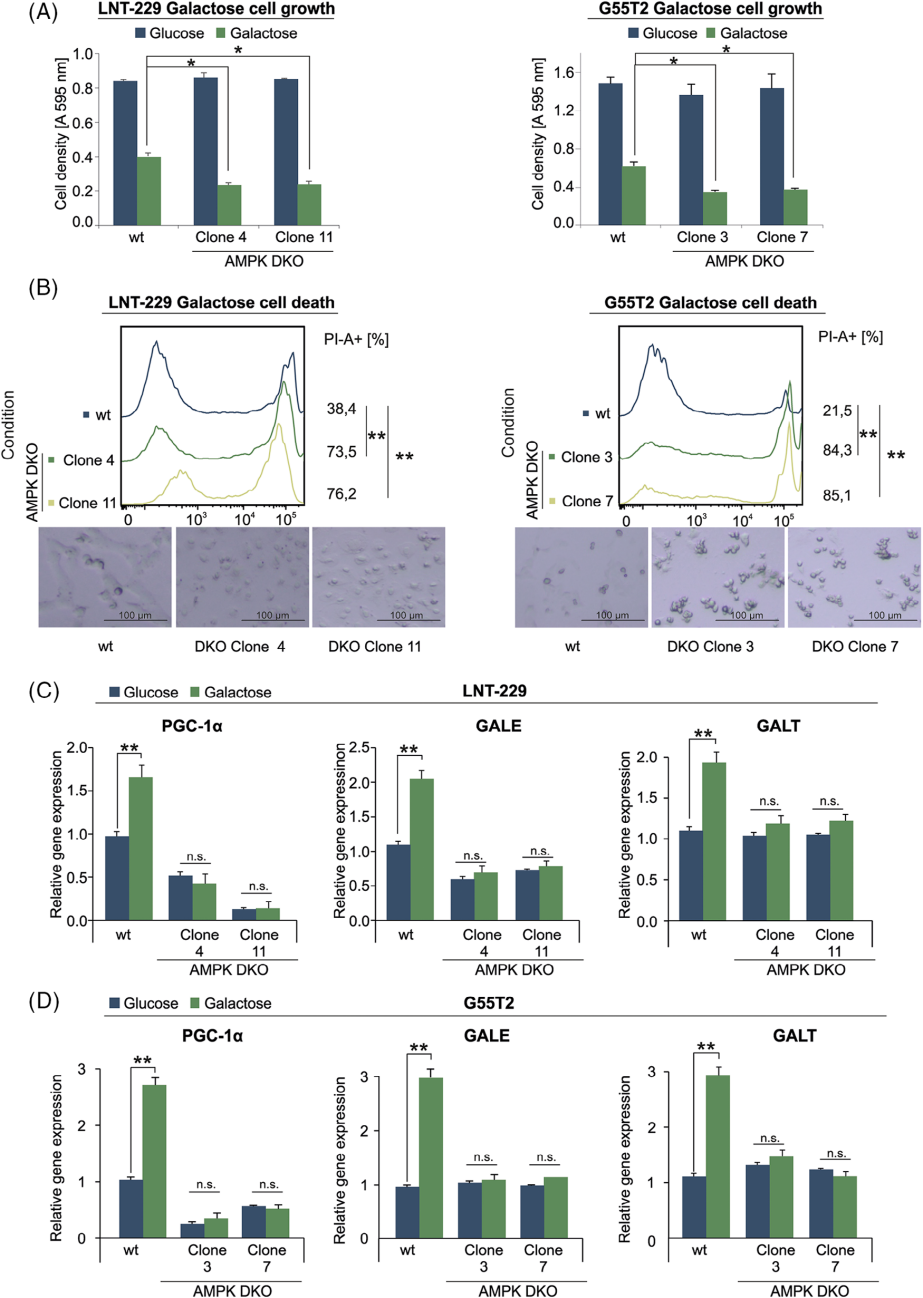
Knockout of AMPK disturbs galactose metabolism. (A, B) LNT‐229 (left panel) and G55T2 (right panel) wt and AMPK DKO cells were incubated in medium containing 25 mM glucose or 25 mM galactose. Cell density was measured by crystal violet staining after 3 days (A). Cell death was determined by propidium iodide staining after 48 h (B). Representative photographs of the cells are included below the panels (bright field microscopy, 48× magnification). (C, D) cDNA of LNT‐229 (upper panel) and G55T2 (lower panel) wt and AMPK‐DKO cells maintained in culture medium with either glucose or galactose for 5 days was generated. Gene expression of PGC‐1α, GALE and GALT was quantified (*n* = 3, mean ± SD, *p < 0.05, **p < 0.01).

**FIGURE 6:**
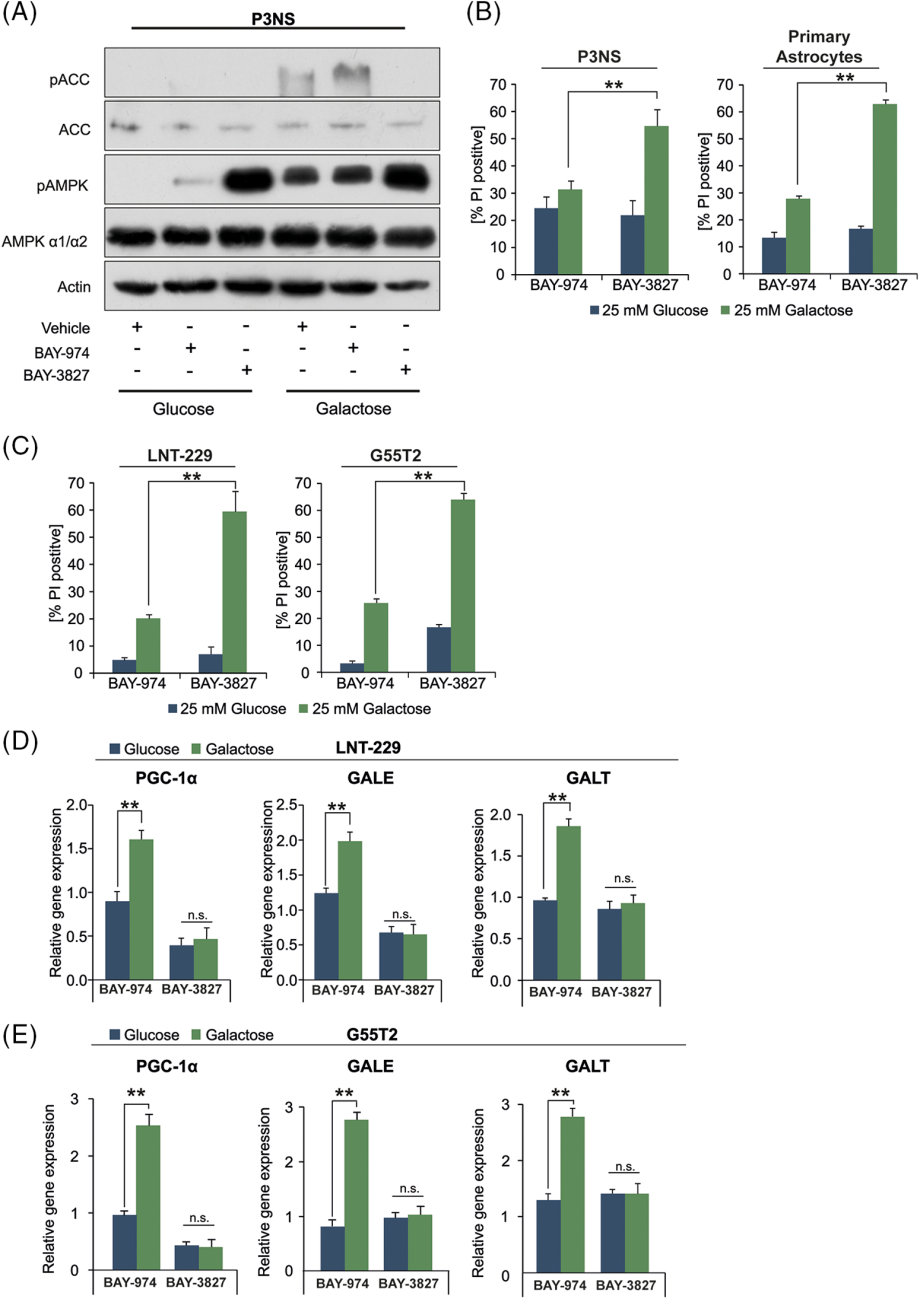
Pharmacological inhibition of AMPK disturbs galactose metabolism in primary cell models. P3NS cells were incubated for 12 h in medium containing either 25 mM glucose or 25 mM galactose and treated with vehicle, 1 µM BAY‐974 and with 1 µM BAY‐3827 as indicated. Cell lysates were analysed by antibodies against ACC, pACC, AMPK, pAMPK and actin. (B, C) P3NS cells and primary human astrocytes (B) as well as LNT‐229 and G55T2 cells (C) were incubated in medium containing either 25 mM glucose or galactose and treated with 1 µM BAY‐974 or 1 µM BAY‐3827. Cell death was quantified by PI staining after 24 h (*n* = 3, mean ± SD, *p < 0.05, **p < 0.01). The percentage of propidium iodide positive cells (PI‐A +) is indicated. (D, E) cDNA of LNT‐229 (D) and G55T2 (E) cells maintained in culture medium with either glucose or galactose for 5 days was generated. Gene expression of PGC‐1α, GALE and GALT was quantified (*n* = 3, mean ± SD, *p < 0.05, **p < 0.01).

To further investigate the significance of AMPK as an adaptive mediator, we used LNT‐229 and G55T2 cells with a double knockout of both catalytic AMPK α1 and α2 subunits (AMPK DKO) (Figure S4A).^16^ As expected, activation of AMPK with A‐769662 only increased protein levels of PGC‐1α in AMPK wt cells and not in AMPK DKO cells (Figure S4B). We asked whether loss of the AMPK‐PGC‐1α axis in the DKO cells would impair their galactose metabolism and decrease their ability to access alternative nutrients. We observed lower levels of cell density in AMPK DKO cells compared to the wild‐type control when grown in galactose containing medium (Figure 5A). Accordingly, incubation with galactose increased the amount of cell death in AMPK DKO cells (Figure 5B) while overexpression of PGC‐1α was able to rescue this effect (Figure S4C and D). When cultivated several days in culture medium with galactose instead of glucose, knockout of AMPK prevented the increase of gene expression levels of PGC‐1α, GALE and GALT in LNT‐229 cells (Figure 5C and D). Gene expression levels for PGC‐1α, GALE, ACAT2 and CPT1c were decreased in LNT‐229 AMPK DKO cells. The expression of ACAT1 and MCAD was not significantly altered by knockout of AMPK (Figure S4E). To investigate the observed effects of AMPK inhibition on galactose metabolism in a primary cell model, we used a novel AMPK inhibitor (BAY‐3827) in primary GB cells P3NS and in primary human astrocytes. BAY‐974 has a similar structure to BAY‐3827 and served as an inactive control compound without affecting AMPK activity.^35^ Treatment with BAY‐3827 increased AMPK phosphorylation but decreased phosphorylation of the established AMPK target ACC compared with the negative control BAY‐974 and vehicle, confirming the inhibitory effect on AMPK (Figure 6A). Simultaneous incubation with galactose and BAY‐3827 resulted in an increased rate of cell death in P3NS cells and in primary human astrocytes (Figure 6B). LNT‐229 and G55T2 cells also displayed higher amounts of cell death when incubated in galactose containing medium with BAY‐3827 (Figure 6C). Treatment with BAY‐3827 also prevented induction of gene expression of PGC‐1α, GALE and GALT in LNT‐229 and G55T2 cells during long‐term culture with galactose (Figure 6D and E). This is in line with the effects observed in AMPK DKO cells (Figure 5B–D).

### 3.6 Tumour cell PGC‐1α expression inversely correlates with hypoxia in human GB tissue

**FIGURE 7:**
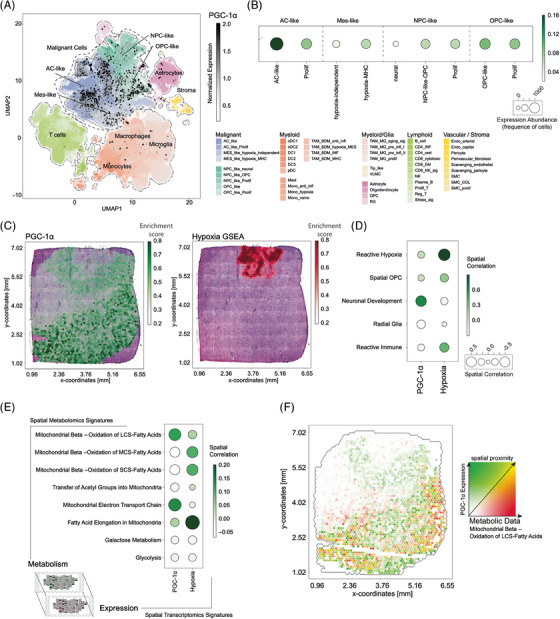
PGC‐1α expression inversely correlates with hypoxia in human GB tissue. (A) GBMap analysis of PGC‐1α‐expression. Colours indicate the cell types and colour hue demonstrate individual cell states. PGC‐1α‐expression is demonstrated as dots in which the intensity is illustrated by shades of grey. (B) Expression of PGC‐1α among annotated cell populations. The size of the circles indicates the numbers of cell that express the gene and the colour indicate the relative expression level. (C) Spatial expression of PGC‐1α based on the spatially resolved transcriptomic dataset along with the histology H&E Images. (D) Spatial correlation of PGC‐1α‐expression and hypoxia gene expression abundance across tumour niches. (E) Multiomic spatial correlation of metabolic signatures (derived from metabolomic data from MALDI) and expression pattern of either PGC‐1α or hypoxia gene set enrichment (from expression data). The size of the circles demonstrates the spatial correlation value. (F) Spatial proximity plots of PGC‐1α expression and long‐chain‐saturated fatty acids metabolism.

To analyse which cell types within the tumour microenvironment express PGC‐1α and whether it is preferentially expressed in tumour cells we analysed the single cell RNA‐seq GBMap dataset. GBMap is a cellular map of GB that integrates multiple scRNA‐seq datasets and is a valuable resource for exploratory analysis, hypothesis generation and testing.^15^, ^36^ We used the annotation level 4 which includes the most detailed annotation of different tumour subtypes and environmental cells (Figure 7A). The GBMap analysis revealed a notable and statistically significant upregulation (*p*_adj _= 1.54 × 10^−6^) of PGC‐1α expression in the astrocyte (AC)‐like subgroup, which is one of the four main cellular states of GBs that have previously been identified by scRNA‐seq.^37^ Additionally, an elevated expression level of PGC‐1α was observed in the OPC‐like cell populations suggesting an association of PGC‐1α to glia like cell populations. Conversely, the expression of PGC‐1α was found to be comparatively low in neuronal‐like cell populations, suggesting a heterogeneous regulation of this gene across distinct cell types within GB (Figure 7B). Moreover, the expression pattern of PGC‐1α was largely confined to tumour cells, exhibiting minimal expression levels in astrocytes or myeloid cells (Figure 7A). Next, we employed spatially resolved transcriptomic data^15^ to evaluate the spatial pattern of PGC‐1α expression and proximity to associated signalling pathways in 16 human GB samples. We started our analysis by examining PGC‐1α expression in GB tissue, identifying a unique pattern that did not coincide with the hypoxia gene set enrichment (Figures 7C and D and S5). This finding aligns with the single cell RNA‐seq analysis, where PGC‐1α expression was not associated with the hypoxia‐associated mesenchymal phenotype or the increased sensitivity of cells overexpressing PGC‐1α to hypoxia‐induced cell death (Figures 7A and B and 2E).^38^ Further we identified high proximity of PGC‐1α expression and spatial niches known as ‘Neuronal Development’ (Figure 7D). These niches have previously been defined and characterised.^15^ The ‘Neuronal Development’ niches in particular represent tumour regions located outside the core and exhibit a substantial distance from the hypoxia core. In contrast, areas characterised by hypoxia displayed a distinct metabolic profile and were associated with niches referred to as ‘Reactive Hypoxia and Reactive Immune’. These findings shed light on the metabolic heterogeneity within the tumour, highlighting the existence of spatially distinct niches with varying metabolic characteristics.

### 3.7 PGC‐1α expression is associated with the fatty acid metabolism and enhanced mitochondrial electron transport chain metabolic signature

In a next step, we aimed to reveal metabolic pathways that positively correlate with expression of PGC‐1α. For this purpose, MALDI (Matrix‐Assisted Laser Desorption/Ionisation) was used to incorporate spatially resolved metabolomic data along with the transcriptomic data. We conducted a spatial correlation analysis between the expression pattern of either PGC‐1α or hypoxia gene expression and several metabolic pathways derived from the MALDI analysis. This allowed us to reveal metabolic processes linked to the expression PGC‐1α. The analysis demonstrated a significant spatial proximity of PGC‐1α with metabolism of long‐chain‐saturated fatty acids (*p*_adj _< 2.2 × 10^−16^) and enhanced mitochondrial electron transport chain (*p*_adj _= 7.39 × 10^−8^) (Figure 7E). Mapping the expression of PGC‐1α and the metabolism long‐chain‐saturated fatty acids in a spatial proximity plot, confirmed the shared spatial pattern (Figure 7F).

### 3.8 PGC‐1α induces proteins of mitochondrial regulation in cell models

For a more global view on the implications of PGC‐1α overexpression, we analysed the proteome of LNT‐229 and G55T2 PGC‐1α overexpressing cells. First, we evaluated the differential protein expression between the PGC‐1α overexpressing cells and the empty vector group (Figure S6A). Interestingly many of the upregulated proteins in the PGC‐1α group were involved in RNA processing and metabolism. Subsequently, we employed the differentially expressed proteins to perform gene set enrichment analysis (GSEA). The resulting network of biological functions revealed distinct signalling communities that were specifically associated with either the PGC‐1α overexpression or the empty vector group (Figure S6B). To address potential biases arising from different cell sources in the analysis of differential gene expression, we employed a network‐based approach called Weighted Gene Co‐expression Network Analysis (WGCNA). WGCNA enabled us to correlate the cell status (overexpression/empty vector) with regulatory modules, providing a means to mitigate any confounding effects and gain a deeper understanding of the underlying biological processes. The correlation analysis between the condition parameter (OE/EV) and protein modules uncovered a noteworthy enrichment of the pink and lightyellow modules, which actively contribute to the observed differences driven by overexpression of PGC‐1α (Figure S6C). However, it is important to note that there are other modules that appear to be specific for certain cell types and, therefore, will not be further investigated in this study. The protein‐protein network we found is visualised by UMAP (Uniform Manifold Approximation and Projection) (Figure S6D). In the next step, we focused on the modules ‘pink’, ‘lightyellow’, ‘brown’ and performed gene set enrichment analysis (Figure S6E) to investigate the biological functions that are upregulated modules correlating with overexpression of PGC‐1α. Most interestingly, the highly upregulated module ‘lightyellow’ also enriches regulatory functions of mitochondria metabolism. We next investigated the proteins in the module more in depth and found numerous proteins that are indeed involved in mitochondrial regulation. A summary of the genes related to of mitochondrial metabolism is provided in Table S1.

## 4 DISCUSSION

Aerobic glycolysis, for a long time, has been considered the energetic driver of cancer cell proliferation in malignant entities including GB. However, this notion has recently been challenged by demonstration that cancer cells may readily metabolise nonglucose nutrients^5^, ^39^ which under glucose deprivation can confer a survival advantage through additional ATP‐generation via oxidative phosphorylation.^40^, ^41^ In breast cancer models, for example, 20% and 80% of produced ATP derived from glycolytic and oxidative metabolism, respectively.^42^ In GB, fatty acids have been reported to be a major contributor to aerobic respiration.^5^ This indicates that tumour cell metabolism is not as uniform as once believed. Particularly in the context of the microenvironment, the ability to dynamically adapt metabolism, that is, metabolic plasticity, is a potentially important determinant of clonal assertiveness with the underlying mechanisms only poorly understood.

In this study, we found that GB cells exposed to alternative nutrient sources during energy stress triggered expression of the respective genes involved in galactose, fatty acid and ketone body metabolism as well as induced oxidative capability (Figure 1D–F). To further study metabolic plasticity in this context, we chose galactose as a model substance that triggers oxidative metabolism^28^, ^32^, ^43^ (Figure 1A–C). In our starvation experiments we specifically chose to exclude glutamine to not have effects distorted, for example, by parallel effects on nitrogen metabolism.^44^ The co‐transcription factor PGC‐1α emerged as a prime candidate for the reprogramming of cellular metabolism (Figure 1D–F). In line, PGC‐1α expression levels were elevated under nonglucose nutrient conditions (Figure 1I and J) and genetic overexpression of PGC‐1α induced oxidative metabolism (Figure 2A and B). Notably, PGC‐1α has previously been shown to have a profound impact on glucose, lipid and ketone body metabolism as well as insulin signalling among several tissue types.^45^, ^46^, ^47^, ^48^

Our proteomic analysis of PGC‐1α overexpressing cells confirmed a positive regulation of pathways of RNA modifying and processing, matching the findings of other groups (Figure S6A and B).^49^ Also, WGCNA analysis revealed an upregulation of proteins involved in mitochondrial metabolism (Figure S6D and E). While GB cells are able to maintain oxidative phosphorylation down to oxygen tensions of 1%,^4^ below this threshold O_2_ consuming pathways are no longer are accessible, turning high PGC‐1α levels into a liability (Figure 2E). This is supported by the spatial distribution pattern of PGC‐1α expression that inversely correlated with hypoxia gene set enrichment (Figure 7C and D and S5). Generally, the GBMap analysis revealed an upregulation of PGC‐1α especially in the AC‐like group, indicating a potential association with astrocytoma‐like characteristics. Only minimal expression levels were detected in astrocytes or myeloid cells implying a specificity to the tumour cell population and underscoring the differential expression across different cell types within the tumour microenvironment (Figure 7A and B). Taken together our observations imply that PGC‐1α is an important factor for cell survival in nutrient scarcity, but needs to be regulated depending on the conditions of the local environment. This could explain why the group with the mixed cell population had the most pronounced tumour growth in the CAM‐assay (Figure S2C and D). It is also plausible that GB cells in the tumour microenvironment organise their metabolism strategically depending on the local condition to support tumour growth as has been demonstrated in ovarian carcinoma models.^50^, ^51^

We hypothesised that a scarcity of glucose as the main nutrient source initially triggered a decline in cellular energy levels which would make AMPK a prime candidate as sensor for alternative nutrient sources to trigger transcriptional upregulation of PGC‐1α. Indeed, we detected elevated levels of phosphorylated AMPK under nonglucose nutrients as a sign of increased activity in our experimental settings (Figure S3C). Pharmacological activation of AMPK with the compound A‐769662 also increased protein expression of PGC‐1α, which could be reverted by inhibition of p38 MAPK (Figure 4A).^9^

It is plausible that loss of AMPK prevents cells both from shutting down ATP consuming pathways and from employing alternative nutrient sources like galactose or fatty acids via PGC‐1α, thus increasing the susceptibility to starvation driven cell death. Compatible with this, we observed decreased cell viability upon exposure to galactose in both AMPK DKO sub cell lines which could be rescued by overexpressing PGC‐1α (Figures 5A and B and S4C and D) as well as increased sensitivity to galactose by pharmacological AMPK inhibition in primary human astrocytes as well as primary glioblastoma P3NS cells (Figure 6B).

While the AMPK‐PGC‐1α axis as a cellular mechanism to cope with metabolic stress has previously found its way into cancer research,^9^, ^52^ its role in GB still remains elusive. To our knowledge, this is the first extensive characterisation of AMPK‐PGC‐1α‐mediated metabolic plasticity in GB. So far, pharmacological AMPK inhibitors were deemed unspecific, which is why we employed GB cells with a double knockout of both catalytic AMPK subunits as well as a novel inhibitor with an inactive control. Our comprehensive analysis of clinical GB samples has revealed a previously unrecognised spatial expression pattern of PGC‐1α. Notably, this pattern is characterised by diminished expression levels of PGC‐1α in areas that exhibit hypoxic features. While further studies are necessary to validate the transcriptomic data on protein level, this novel insight nevertheless contributes to our understanding of how local microenvironmental conditions might influence gene expression profiles and cellular metabolism, particularly in terms of energy regulation and mitochondrial function, where PGC‐1α plays a critical role. It is also critical to acknowledge, that PGC‐1α‐activity is not only regulated by expression levels, but also by post‐translational modifications like direct phosphorylation by AMPK.^53^

**FIGURE 8:**
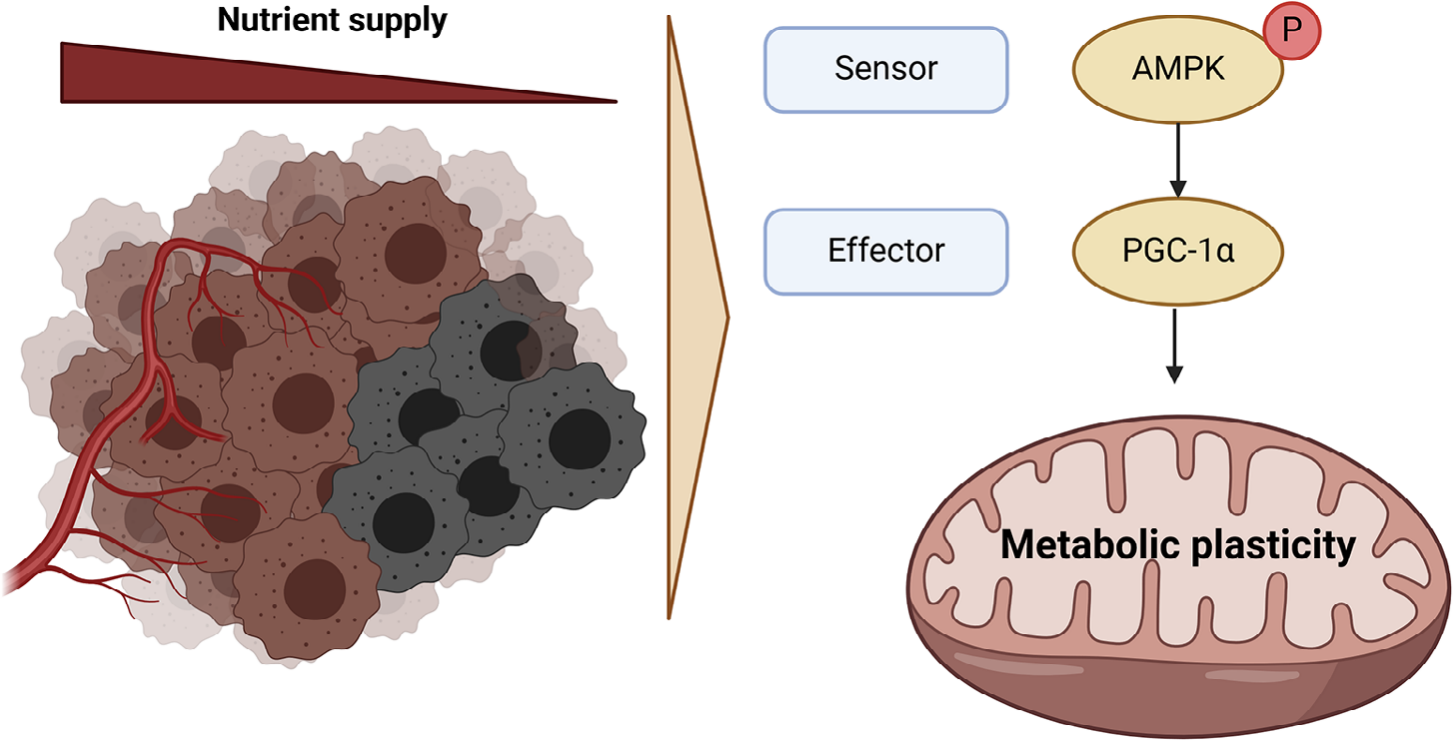
Schematic drawing illustrating the mechanism of metabolic plasticity via AMPK‐dependent activation of PGC‐1α during nutrient deficiency in glioblastoma (GB). Created in BioRender (BioRender.com/m13m543).

Our results identify PGC‐1α as an important orchestrator of metabolic plasticity that allows tumour cells to switch between different nutrient scenarios. From a clinical point of view, both, high and low expression levels of PGC‐1α, have been associated with bad prognosis depending on the cancer entity, illustrating its paradoxical role.^13^, ^54^, ^55^ The effector mechanism of PGC‐1α to reprogram cellular metabolism by promoting mitochondrial metabolism in response to external stressors makes it a plausible mediator of therapy‐resistance and tumour cell survival and therefore a promising target for therapeutic intervention. This is supported by our previous work in which we demonstrated that knockdown of PGC1‐α lead to a less aggressive tumour‐phenotype in a murine model. However we also reported that under hypoxic conditions knockdown of PGC‐1α can also induce a therapeutically detrimental resistance against hypoxia.^12^ Therefore, especially in tumours with frequently occurring hypoxia like GB, innovative treatment algorithms, like sequentially targeting tumour cells in different metabolic niches are promising approaches. A conceivable algorithm would be to first target oxidative cells within the tumour (e.g. by inhibition of PGC‐1α or of oxidative metabolism) and then, in a second step, to exploit specific vulnerabilities of remaining, for example, hypoxic cells. For this purpose, inhibitors of HIF1 or the Unfolded Protein Response (UPR) could be interesting candidates. Targeting PGC‐1α in GB may be challenging. Using the previously published PGC‐1α inhibitor SR‐18292 (data not shown), we did not observe relevant effects in GB cell lines which contrasts published data demonstrating the inhibitor's efficacy in other models, such as cholangiocarcinoma cell models,^56^ suggesting a differential regulation in GB. Our results highlight the potential and complexity of inhibiting metabolic adaptive responses as novel tumour therapies. The mechanism of AMPK‐mediated PGC‐1α activation to utilise alternative nutrients described here (Figure 8) may be a promising new candidate for such a metabolic targeted therapy.

## AUTHOR CONTRIBUTIONS

Benedikt Sauer, Nadja I. Lorenz, Johannes Rieger, Joachim P. Steinbach, Dieter Henrik Heiland and Michael W. Ronellenfitsch conceptualised this study. Benedikt Sauer, Nadja I. Lorenz, Süleyman Bozkurt, Dorothea Schulte, Hans Urban, Mohaned Benzarti, Johannes Meiser, Patrick N. Harter, Christian Münch and Michael W. Ronellenfitsch performed and helped with the experiments. Benedikt Sauer, Jan Kueckelhaus, Süleyman Bozkurt, Mohaned Benzarti, Hans Urban, Giulia Villa, Christian Münch and Dieter Henrik Heiland performed bioinformatics and statistical data analysis. Benedikt Sauer prepared the figures. All authors contributed to data interpretation. Benedikt Sauer, Dieter Henrik Heiland and Michael W. Ronellenfitsch wrote the original draft of the manuscript. All authors revised the manuscript and approved of its final version.

## CONFLICT OF INTEREST STATEMENT

JPS reports honoraria for lectures or advisory board participation or consulting or travel grants from Abbvie, Roche, Boehringer, Bristol‐Myers Squibb, Medac, Mundipharma and UCB. Michael W. Ronellenfitsch reports a research grant from UCB as well as honoraria for advisory board participation from Alexion. All other authors declare no conflicts of interest.

## ETHICS STATEMENT

The experimental design and data valuation derived from human material has been approved by the local ethics committee of the University of Freiburg (protocol 100020/09 and 472/15_160880)

## Supporting information



Figure S1. (A) LNT‐229 cells were exposed to either glucose or galactose containing (2 mM) serum‐free medium under normoxic conditions or 0.1% oxygen. Cell death was quantified by LDH release after 18 h (*n* = 4, mean ± SD, *p < 0.05, **p < 0.01). (B) LNT‐229 and G55T2 pcDNA3 PGC‐1α and control cells (empty vector control, EV), and LNT‐229 pTetOne PGC‐1α cells with and without 0.1 µg/mL doxycycline were analysed by qPCR. PGC‐1α overexpression was confirmed (*n* = 3, mean ± SD). (C) Representative immunoblot showing overexpression of PGC‐1α in the cells mentioned in (A) and knockout (KO) of PGC‐1α in HAP1 cells. LN‐319 cells were used as a positive control, SKMEL‐28 cells were used as a negative control. (D, F) cDNA of LNT‐229 and G55T2 EV and PGC‐1α cells and LNT‐229 pTetOne PGC‐1α cells with and without 0.1 µg/mL doxycycline cultured in serum‐free medium for 24 h was generated. Gene expression of MT‐CYB (D), SOD1 and SOD2 (F) quantified, values are normalised to 18S as well as SDHA housekeeping gene expression (*n* = 3, mean ± SD, *p < 0.05, **p < 0.01). (E) ROS levels were measured by H2DCFDA‐FACS (*n* = 3, mean ± SD, *p < 0.05. (G) Cells were kept in glucose restricted (2 mM glucose) serum‐free DMEM under normoxic (21%) and hypoxic (0.1%) conditions for 6 h. Remaining Glucose was measured in the supernatant (*n* = 3, mean ± SD). (H) LNT‐229 and G55T2 EV and PGC‐1α cells were incubated in serum‐free medium without glutamine for 6 h. Intracellular ATP levels relative to the empty vector control were determined.

Figure S2. Fertilised chicken eggs were incubated at 37°C and high humidity for 7 days before inoculation with tumour cells. G55T2 EV, G55T2 PGC‐1α cells, and a 1:1 mixture of both groups were used. (A) Open situs at day 7. (B) The experiments were stopped after another 7 days of incubation of the eggs and the tumour was isolated. (C) Visual representation of tumour size of the different groups. (D) Tumour weight was determined using a fine balance. (E) LNT‐229 and G55T2 EV and PGC‐1α cells and LNT‐229 pTetOne PGC‐1α cells with and without 0.1 µg/mL doxycycline were exposed to 25 mM galactose and to 2 mM glucose with the addition of 100 µM linoleic acid or 5 mM 3OHB in hypoxia (1% oxygen). Cell density was measured by crystal violet staining after 3 days (*n* = 3, mean ± SD).

Figure S3. (A) LNT‐229 (left panel) and G55T2 (right panel) EV and PGC‐1α cells were incubated in medium containing 2 mM glucose with or without addition of 5 mM 3OHB. Oxygen consumption was measured by a fluorescence‐based assay (*n* = 3, mean, **p < 0.01). (B) LNT‐229 (upper panel) and G55T2 (lower panel) EV and PGC‐1α cells were incubated in medium containing 2 mM glucose with or without addition of 100 µM linoleic acid and treated with vehicle or 100 µM etomoxir. Oxygen consumption was measured by a fluorescence‐based assay (*n* = 3, mean, **p < 0.01). Data for EV cells are presented in the left panels, for the PGC‐1α cells in the right panels. (C) LNT‐229 and G55 cells were incubated for 12 h in medium as indicated. Cellular lysates were analysed by immunoblot with antibodies for PGC‐1α, Phospho‐AMPKα (Thr172), AMPKα and actin.

Figure S4. (A) Representative immunoblot of α1 and 2 subunits of AMPK and actin in LNT‐229 (left panel) and G55T2 (right panel) wild‐type (wt) and AMPK DKO cells. (B) G55T2 and LNT‐229 wt and AMPK DKO cells were incubated for 24 h in serum‐free medium and treated with 100 µM A‐769662 as indicated. Cellular lysates were analysed by immunoblot with antibodies for PGC‐1α, Phospho‐AMPKα (Thr172), AMPKα and actin. (C, D) LNT‐229 AMPK DKO cells with and without overexpression of PGC‐1α were incubated in medium containing 25 mM galactose. Cell density was measured by crystal violet staining after 24 h (*n* = 3, mean ± SD, p < 0.05) (C). Cell death was determined by propidium iodide staining after 24 h. The percentage of propidium iodide positive cells is indicated (n = 3, mean, *p < 0.05) (D). (E) cDNA from LNT‐229 wt and AMPK DKO cells cultured in serum‐free medium for 24 h was generated. Gene expression of PGC‐1α, GALE, ACAT1, ACAT2, MCAD and CPT1c was quantified. (*n* = 3, mean ± SD, *p < 0.05, **p < 0.01).

Figure S5. Representative examples of spatial transcriptomic correlation of PGC‐1α gene expression (in green) and hypoxia gene set enrichment (in red) in human GB samples.

Figure S6. (A) Heatmap of top differentially expressed proteins between PGC‐1α overexpressing and control cells. (B) GSEA of differentially expressed proteins. (C) Depiction of distinct modules identified by WGCNA analysis associated with overexpression of PGC‐1α and control. The *p*‐value threshold (adjusted for multiple testing) was set to *p*_adj _< 0.05. All correlations which did not reach the significance level were marked with ‘X’. (D) Reduced‐dimensional visualisation of the protein‐protein network by UMAP‐analysis. (E) GSEA of the modules ‘pink’, ‘lightyellow’, ‘brown’ that are correlating with overexpression of PGC‐1α.

Supporting Information

## Data Availability

The spatial transcriptomic data and MALDI data have been published previously^15^ and are deposited and publicly available at Datadryad (https://doi.org/10.5061/dryad.h70rxwdmj). The mass spectrometry proteomics data have been deposited to the ProteomeXchange Consortium via the PRIDE^54^ partner repository with the dataset identifier PXD046679. All other datasets used in the current study are available from the corresponding author on reasonable request.
