# Supplementary figures and images for: An AMP‐activated protein kinase‐PGC‐1α axis mediates metabolic plasticity in glioblastoma
Source: Clin Transl Med. 2024 Nov 17;14(11):e70030. doi: 10.1002/ctm2.70030 (PMC11570551; doi:10.1002/ctm2.70030)

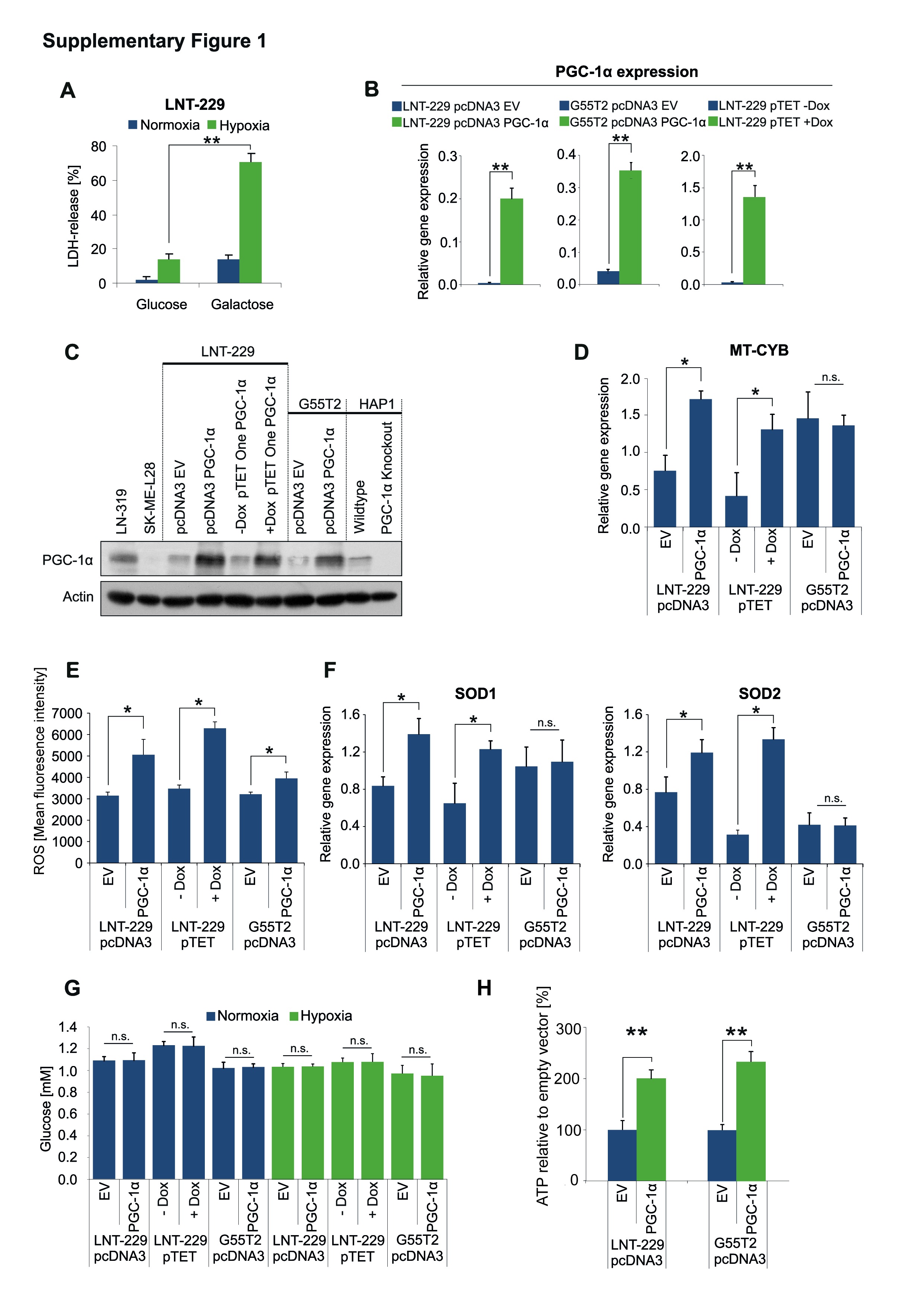

Supplement: Supplementary file 1 — Figure S1. (A) LNT‐229 cells were exposed to either glucose or galactose containing (2 mM) serum‐free medium under normoxic conditions or 0.1% oxygen. Cell death was quantified by LDH release after 18 h (n = 4, mean ± SD, *p < 0.05, **p < 0.01). (B) LNT‐229 and G55T2 pcDNA3 PGC‐1α and control cells (empty vector control, EV), and LNT‐229 pTetOne PGC‐1α cells with and without 0.1 µg/mL doxycycline were analysed by qPCR. PGC‐1α overexpression was confirmed (n = 3, mean ± SD). (C) Representative immunoblot showing overexpression of PGC‐1α in the cells mentioned in (A) and knockout (KO) of PGC‐1α in HAP1 cells. LN‐319 cells were used as a positive control, SKMEL‐28 cells were used as a negative control. (D, F) cDNA of LNT‐229 and G55T2 EV and PGC‐1α cells and LNT‐229 pTetOne PGC‐1α cells with and without 0.1 µg/mL doxycycline cultured in serum‐free medium for 24 h was generated. Gene expression of MT‐CYB (D), SOD1 and SOD2 (F) quantified, values are normalised to 18S as well as SDHA housekeeping gene expression (n = 3, mean ± SD, *p < 0.05, **p < 0.01). (E) ROS levels were measured by H2DCFDA‐FACS (n = 3, mean ± SD, *p < 0.05. (G) Cells were kept in glucose restricted (2 mM glucose) serum‐free DMEM under normoxic (21%) and hypoxic (0.1%) conditions for 6 h. Remaining Glucose was measured in the supernatant (n = 3, mean ± SD). (H) LNT‐229 and G55T2 EV and PGC‐1α cells were incubated in serum‐free medium without glutamine for 6 h. Intracellular ATP levels relative to the empty vector control were determined. [file CTM2-14-e70030-s003.tif]

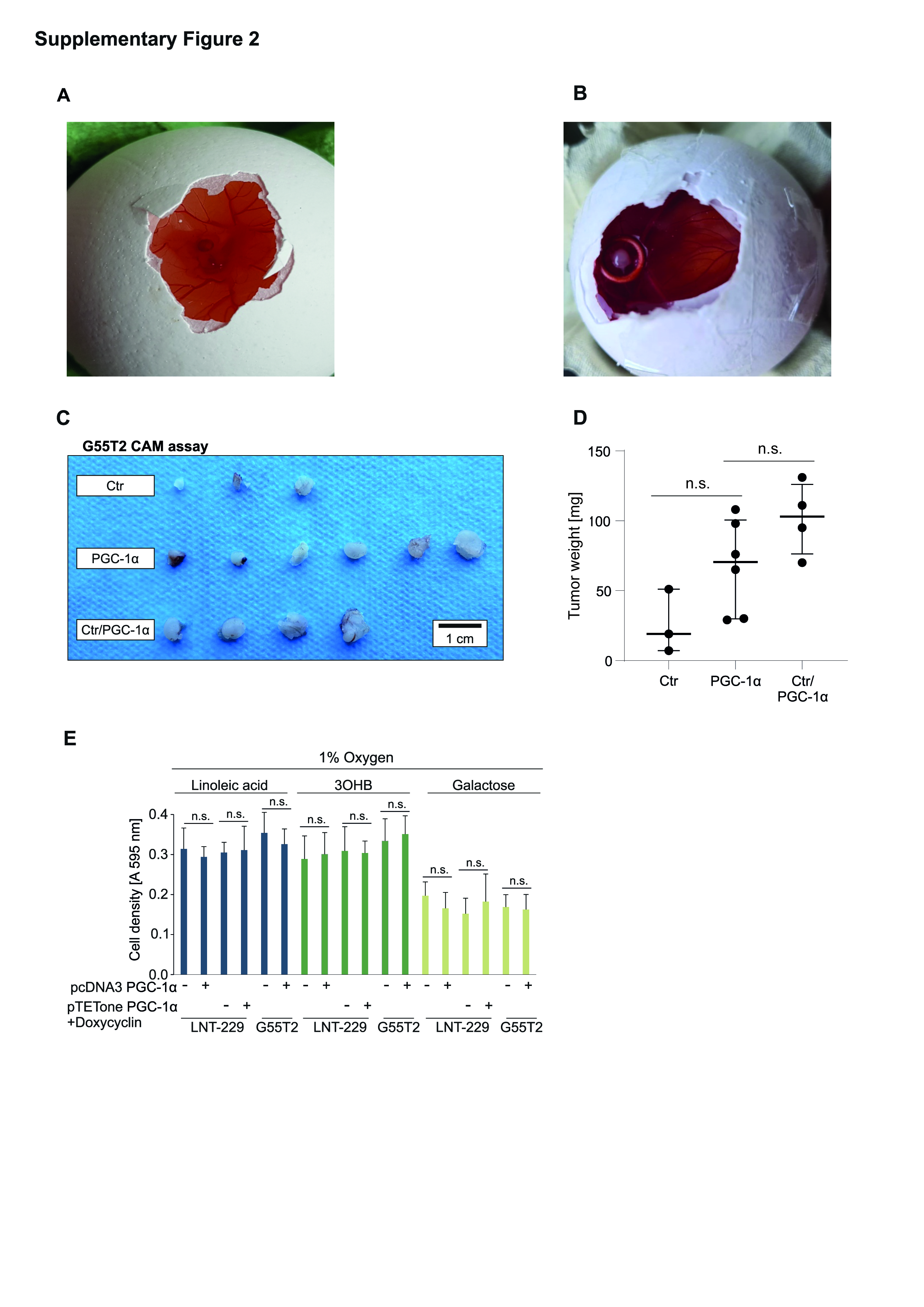

Supplement: Supplementary file 2 — Figure S2. Fertilised chicken eggs were incubated at 37°C and high humidity for 7 days before inoculation with tumour cells. G55T2 EV, G55T2 PGC‐1α cells, and a 1:1 mixture of both groups were used. (A) Open situs at day 7. (B) The experiments were stopped after another 7 days of incubation of the eggs and the tumour was isolated. (C) Visual representation of tumour size of the different groups. (D) Tumour weight was determined using a fine balance. (E) LNT‐229 and G55T2 EV and PGC‐1α cells and LNT‐229 pTetOne PGC‐1α cells with and without 0.1 µg/mL doxycycline were exposed to 25 mM galactose and to 2 mM glucose with the addition of 100 µM linoleic acid or 5 mM 3OHB in hypoxia (1% oxygen). Cell density was measured by crystal violet staining after 3 days (n = 3, mean ± SD). [file CTM2-14-e70030-s002.tif]

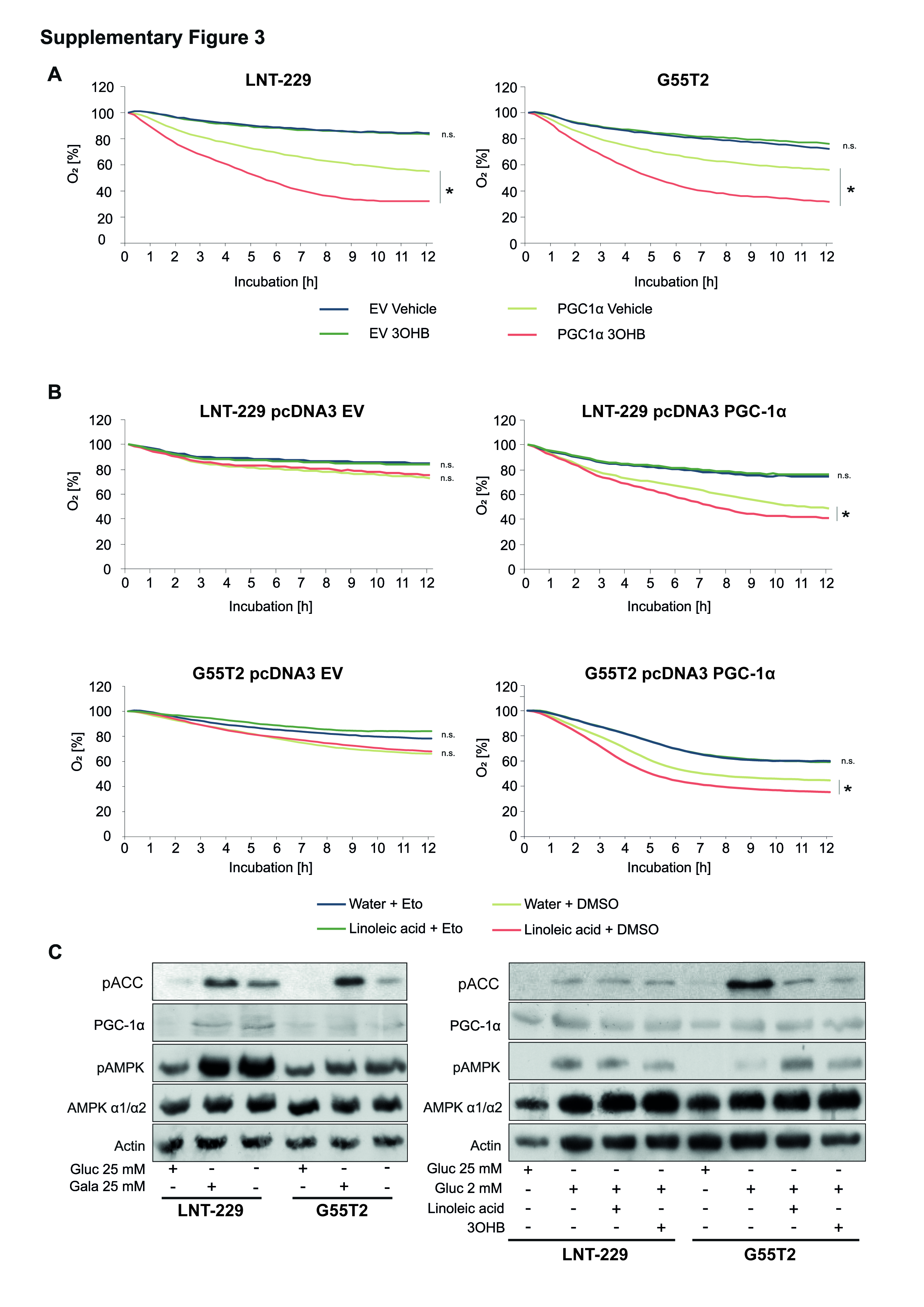

Supplement: Supplementary file 3 — Figure S3. (A) LNT‐229 (left panel) and G55T2 (right panel) EV and PGC‐1α cells were incubated in medium containing 2 mM glucose with or without addition of 5 mM 3OHB. Oxygen consumption was measured by a fluorescence‐based assay (n = 3, mean, **p < 0.01). (B) LNT‐229 (upper panel) and G55T2 (lower panel) EV and PGC‐1α cells were incubated in medium containing 2 mM glucose with or without addition of 100 µM linoleic acid and treated with vehicle or 100 µM etomoxir. Oxygen consumption was measured by a fluorescence‐based assay (n = 3, mean, **p < 0.01). Data for EV cells are presented in the left panels, for the PGC‐1α cells in the right panels. (C) LNT‐229 and G55 cells were incubated for 12 h in medium as indicated. Cellular lysates were analysed by immunoblot with antibodies for PGC‐1α, Phospho‐AMPKα (Thr172), AMPKα and actin. [file CTM2-14-e70030-s005.tif]

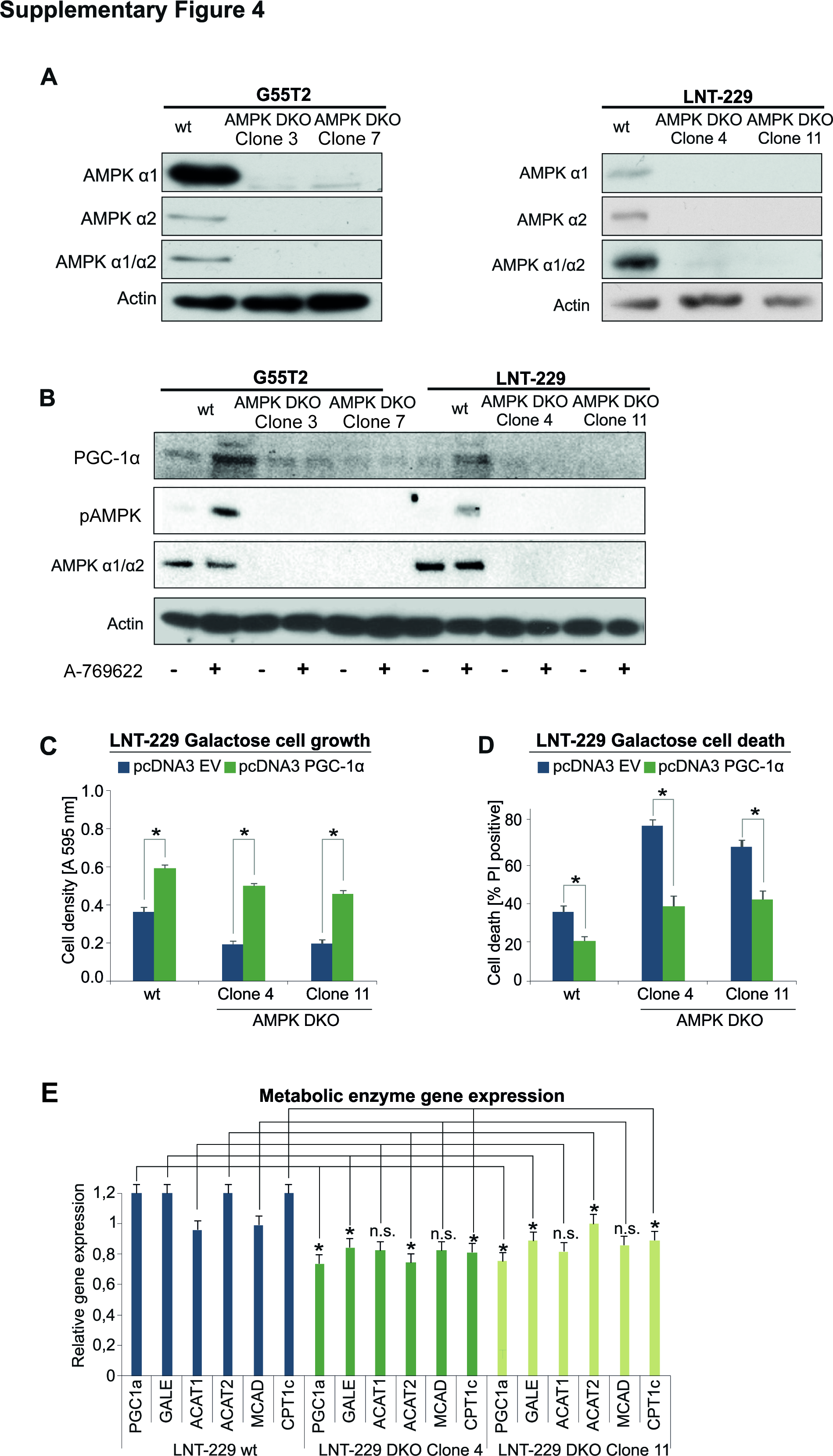

Supplement: Supplementary file 4 — Figure S4. (A) Representative immunoblot of α1 and 2 subunits of AMPK and actin in LNT‐229 (left panel) and G55T2 (right panel) wild‐type (wt) and AMPK DKO cells. (B) G55T2 and LNT‐229 wt and AMPK DKO cells were incubated for 24 h in serum‐free medium and treated with 100 µM A‐769662 as indicated. Cellular lysates were analysed by immunoblot with antibodies for PGC‐1α, Phospho‐AMPKα (Thr172), AMPKα and actin. (C, D) LNT‐229 AMPK DKO cells with and without overexpression of PGC‐1α were incubated in medium containing 25 mM galactose. Cell density was measured by crystal violet staining after 24 h (n = 3, mean ± SD, p < 0.05) (C). Cell death was determined by propidium iodide staining after 24 h. The percentage of propidium iodide positive cells is indicated (n = 3, mean, *p < 0.05) (D). (E) cDNA from LNT‐229 wt and AMPK DKO cells cultured in serum‐free medium for 24 h was generated. Gene expression of PGC‐1α, GALE, ACAT1, ACAT2, MCAD and CPT1c was quantified. (n = 3, mean ± SD, *p < 0.05, **p < 0.01). [file CTM2-14-e70030-s004.tif]

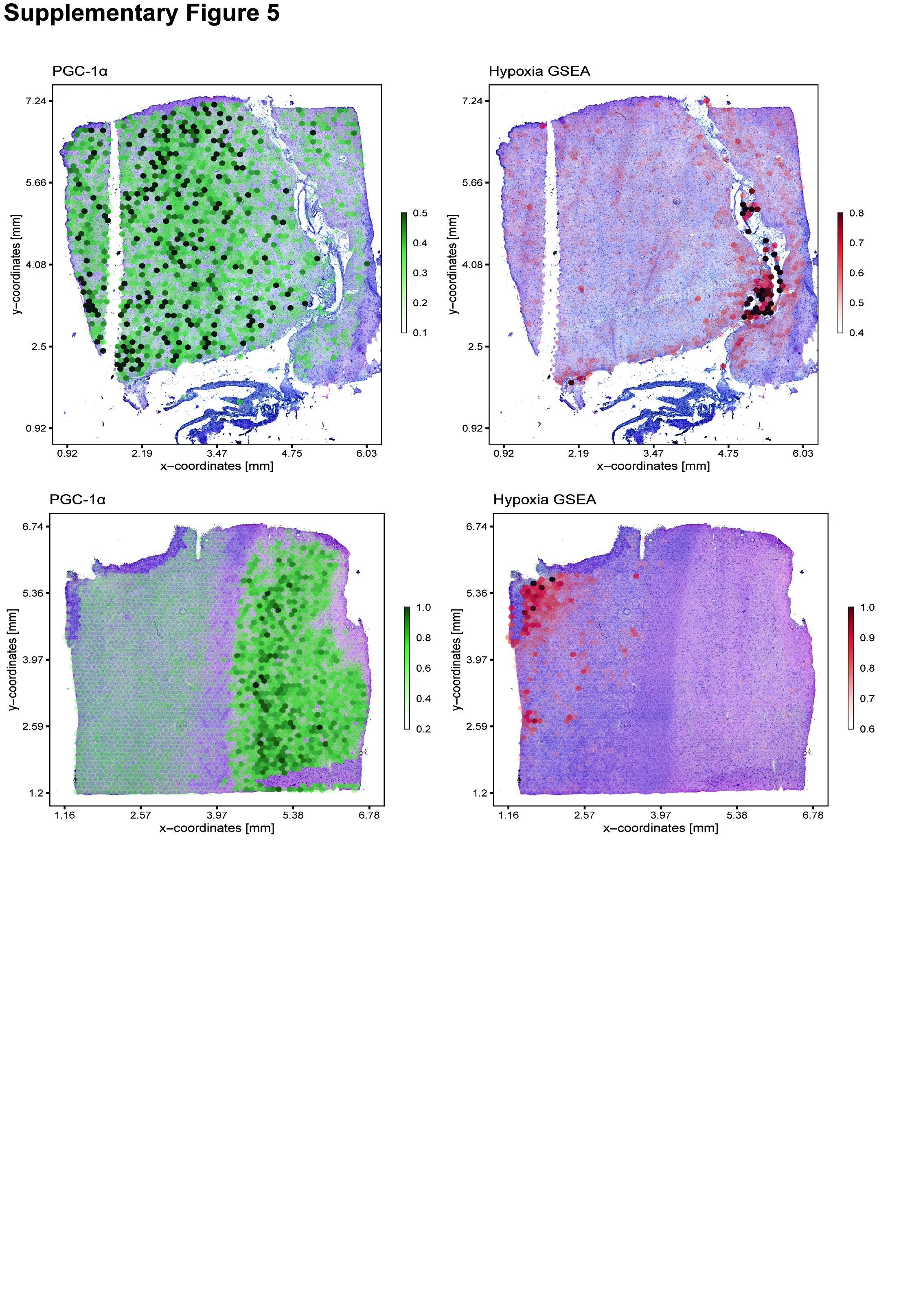

Supplement: Supplementary file 5 — Figure S5. Representative examples of spatial transcriptomic correlation of PGC‐1α gene expression (in green) and hypoxia gene set enrichment (in red) in human GB samples. [file CTM2-14-e70030-s007.tif]

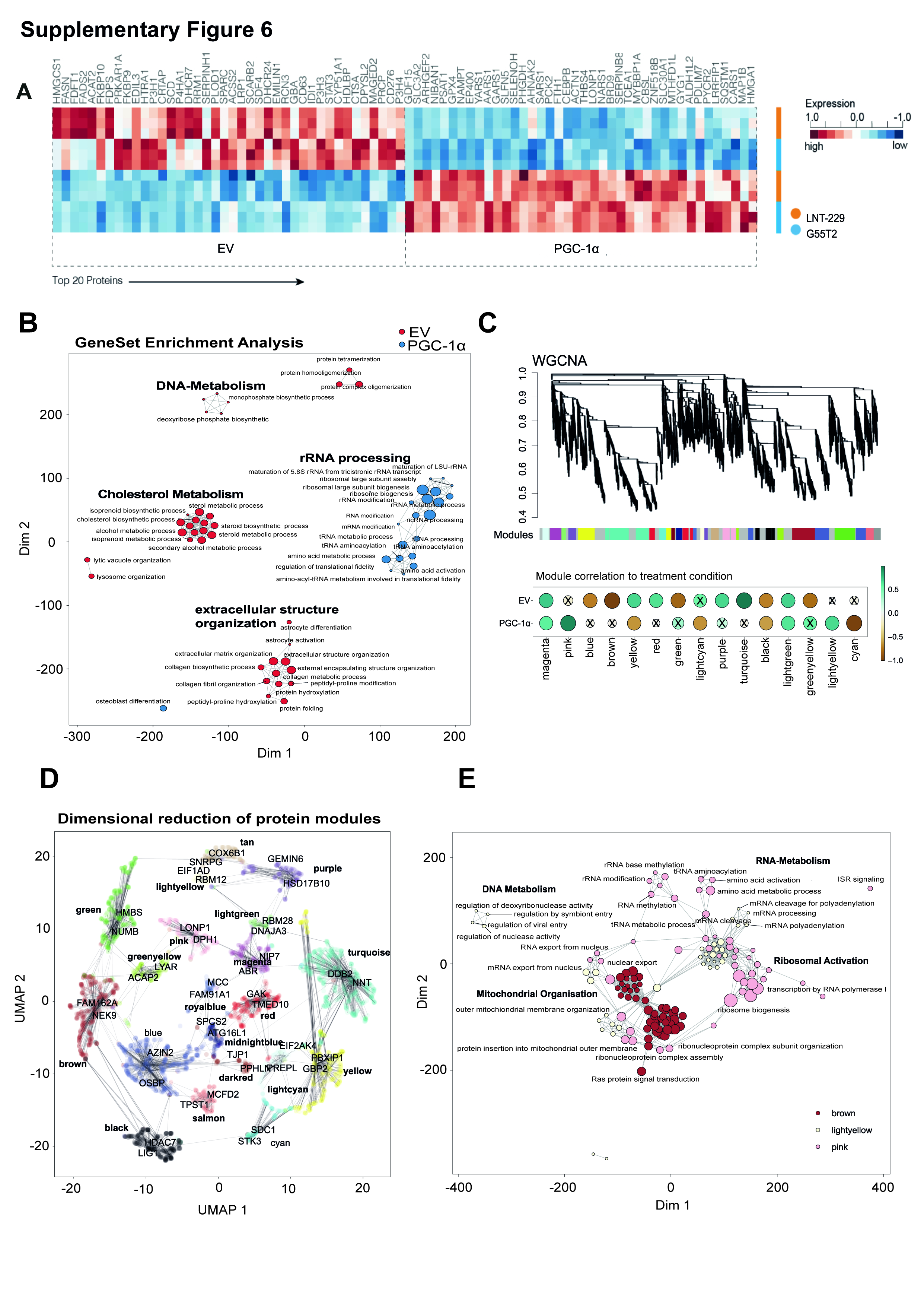

Supplement: Supplementary file 6 — Figure S6. (A) Heatmap of top differentially expressed proteins between PGC‐1α overexpressing and control cells. (B) GSEA of differentially expressed proteins. (C) Depiction of distinct modules identified by WGCNA analysis associated with overexpression of PGC‐1α and control. The p‐value threshold (adjusted for multiple testing) was set to p adj < 0.05. All correlations which did not reach the significance level were marked with ‘X’. (D) Reduced‐dimensional visualisation of the protein‐protein network by UMAP‐analysis. (E) GSEA of the modules ‘pink’, ‘lightyellow’, ‘brown’ that are correlating with overexpression of PGC‐1α. [file CTM2-14-e70030-s001.tif]
