# Supplementary material for: An AMP‐activated protein kinase‐PGC‐1α axis mediates metabolic plasticity in glioblastoma
Source: Clin Transl Med. 2024 Nov 17;14(11):e70030. doi: 10.1002/ctm2.70030 (PMC11570551; doi:10.1002/ctm2.70030)
Supplement: Supplementary file 7 — Supporting Information [file CTM2-14-e70030-s006.docx]

**Supplementary Materials and Methods**

**Reagents, cell lines and culture conditions**

All reagents not specified were purchased from Sigma (Darmstadt, Germany). Etomoxir was purchased from Cayman chemicals (Ann Arbor, Michigan, USA) and A-769662 from Tocris (Bristol, United Kingdom). The AMPK inhibitor BAY-3827 and the corresponding inactive control compound BAY-974 were obtained from the "Donated Probes Set" of the Structural Genomics Consortium (SGC) and were and were provided to us by Prof. Stefan Knapp (Institute of Pharmaceutical Chemistry, Goethe University Frankfurt, Germany) (1). LNT-229 and LN-319 cells were a kind gift of Dr. N. de Tribolet (Lausanne, Switzerland) (2,3). LNT-229 cells were authenticated via STR analysis by Multiplexion (Heidelberg, Germany). The STR profile of the LNT-229 cells matched with the known profile for LN-229, LNT-229 cells only differ from LN-229 cells by their p53 status (LNT-229 are *TP53* wildtype) (4). G55T2 cells were a kind gift of Manfred Westphal and Kathrin Lamszus (Hamburg, Germany) (5). The human haploid cell line HAP1 wt (derived from the leukemia cell line KBM-7) and CRISPR/Cas9 PGC1-α KO cells were purchased from Horizon Discovery (Waterbeach, United Kingdom). SK-MEL-28 cells have previously been described (6). Cell lines were maintained at 37 °C in a cell culture incubator (Binder, Tuttlingen, Germany) under a CO_2_ atmosphere (5%) in DMEM containing 10% FCS (Biochrom KG, Berlin, Germany), 100 IU/mL penicillin, and 100 µg/mL streptomycin (Life Technologies, Karlsruhe, Germany). The primary human astrocytes were purchased from Innoprot (Derio, Spain) and cultured in astrocyte medium containing 10% FCS, 100 IU/mL penicillin, and 100 µg/mL streptomycin and 5 ml Astrocyte Growth Supplement (AGS). P3NS primary GB cells derived from a murine orthotopic primary patient-derived xenograft model and were provided by Simone Niclou (Val Fleuri, Luxembourg) (7). P3NS cells were cultured in serum-free neurobasal medium (Life Technologies) supplemented with B27 (Life Technologies, Carlsbad, California, USA), 2 mM L-glutamine, 30 IU/ml penicillin-streptomycin, 1 IU/ml heparin, 20 ng/ml bFGF, and 20 ng/ml EGF (ReliaTech, Wolfenbüttel, Germany). Subculturing was performed at 90% confluence for all cells. For experimental conditions where glucose-free medium or distinct glucose conditions were required, glucose-free DMEM was used and the desired amount of glucose was supplemented accordingly. FCS was omitted in all experiments. When comparing Rho0 and Rho+ cells, uridine was supplemented because Rho0 cells are defective in pyrimidine synthesis and require uridine for proliferation.

**Mass Spectrometry sample preparation and liquid chromatography MS**

**Sample preparation for mass spectrometry**

Lysates precipitation was performed with methanol/chloroform. Afterwards, proteins were resuspended in 8 M Urea/10 mM EPPS pH 8.2. Concentration of proteins was determined by Bradford assay and 300 µg of protein per sample was digested by diluting to 1 M Urea with 10 mM EPPS pH 8.2 and overnight incubation at 37 ^o^C with LysC (Wako Chemicals, Neuss, Germany) at 1:50 (w/w) ratio and Trypsin (Promega, Madison, WI, USA, V5113) at 1:100 (w/w) ratio. Digests were acidified using TFA and tryptic peptides were purified by tC18 SepPak (50 mg, Waters WAT054955, Milford, MA, USA). Peptide concentrations were determined with a μBCA assay (ThermoFisher Scientific, 23235) and 25 µg peptides per sample were labeled with TMTpro reagents (Thermo Scientific, A44520). TMT labeled samples were normalized after a single injection measurement by LC-MS/MS to equimolar ratios for each channel. Labeled peptide samples were pooled, fractionated into 8 fractions using the High pH Reversed-Phase Peptide Fractionation Kit (ThermoFisher Scientific 84868) according to the manufacturer protocol and dried.

**Liquid chromatography mass spectrometry (LC-MS^3^)**

All mass spectrometry data was acquired in centroid mode on an Orbitrap Fusion Lumos mass spectrometer hyphenated to an easy-nLC 1200 nano HPLC system using a nanoFlex ion source (ThermoFisher Scientific, Waltham, MA, USA). Peptides were separated on a self-made, 22 cm long, 75 µm ID fused-silica column, packed in house with 1.9 µm C18 particles (ReproSil-Pur, Dr. Maisch, Ammerbuch-Entringen, Germany) and heated to 50°C using an integrated column oven (Sonation, Biberach, Germany). HPLC solvents consisted of 0.1% Formic acid in water (Buffer A) and 0.1% Formic acid, 80% acetonitrile in water (Buffer B).

For proteome analysis, a synchronous precursor selection (SPS) multi-notch MS3 method was used in order to minimize ratio compression as previously described (8). Individual peptide fractions were eluted by a non-linear gradient adapted for each fraction (F1: 3-24, F2: 4-26, F3: 5-28, F4: 6-30, F5: 7-32, F6: 8-34, F7: 9-36, F8: 10-38 % B) over 150 minutes followed by a step-wise increase to 95% B in 6 minutes which was held for another 9 minutes. Full scan MS spectra (350-1400 m/z) were acquired with a resolution of 120 000 at m/z 200, maximum injection time of 100 ms and AGC target value of 4 x 10^5^. The 10 most intense precursors with a charge state between 2 and 5 per full scan were selected for fragmentation (“Top 10”) and isolated with a quadrupole isolation window of 0.7 Th. MS2 scans were performed in the Ion trap (Turbo) using a maximum injection time of 50 ms, AGC target value of 1.5 x 10^4^ and fragmented using CID with a normalized collision energy (NCE) of 35%. SPS-MS3 scans for quantification were performed on the 10 most intense MS2 fragment ions with an isolation window of 0.7 Th (MS) and 2 m/z (MS2). Ions were fragmented using HCD with an NCE of 50% and analyzed in the Orbitrap with a resolution of 50,000 at m/z 200, scan range of 110-500 m/z, AGC target value of 1.5 x10^5^ and a maximum injection time of 86 ms. Repeated sequencing of already acquired precursors was limited by setting a dynamic exclusion of 45 seconds and 7 ppm and advanced peak determination was deactivated.

**Proteomics data analysis**

Raw data was analyzed with Proteome Discoverer 2.4 (ThermoFisher Scientific). SequenceHT node was selected for database searches of MS2-spectra. Human trypsin digested proteome (Homo sapiens SwissProt database (TaxID:9606, version 12 March 2020)) was used for protein identifications. Contaminants (MaxQuant “contamination.fasta”) were determined for quality control. TMTpro (+304.207) at the N-terminus, TMTpro (K, +304.207) at lysine and carbamidomethyl (C, +57.021) at cysteine residues were set as fixed modifications. Methionine oxidation (M, +15.995) and acetylation (+42.011) at the protein N-terminus were set for dynamic modifications. Precursor mass tolerance was set to 7 ppm and fragment mass tolerance was set to 0.5 Da. Default percolator settings in PD were used to filter perfect spectrum matches (PSMs). Reporter ion quantification was achieved with default settings in consensus workflow. Protein file from PD was then exported to Excel for further processing. Normalized abundances from protein file were used for statistical analysis after contaminations and complete empty values were removed. Significantly altered proteins were determined by a two-sided, unpaired Student’s t-tests (p-value < 0.05), adding minimum log2 fold-change cut-off (≥ 0.5) with R version 4.0.2 (9) in RStudio (10).

**References**

1. Lemos C, Schulze VK, Bader B, et al. Abstract 5873: BAY-3827, a selective inhibitor of AMPK for the evaluation of the role of AMPK in Myc-dependent tumors. In: Experimental and Molecular Therapeutics: American Association for Cancer Research; 07012018. p.5873.

2. Studer A, Tribolet N de, Diserens AC, et al. Characterization of four human malignant glioma cell lines. Acta Neuropathol. 1985;66(3):208–17. doi:10.1007/BF00688585 Cited in: PubMed; PMID 2990147.

3. Ronellenfitsch MW, Brucker DP, Burger MC, et al. Antagonism of the mammalian target of rapamycin selectively mediates metabolic effects of epidermal growth factor receptor inhibition and protects human malignant glioma cells from hypoxia-induced cell death. Brain. 2009;132(Pt 6):1509–22. doi:10.1093/brain/awp093 Cited in: PubMed; PMID 19416948.

4. Wischhusen J, Naumann U, Ohgaki H, et al. CP-31398, a novel p53-stabilizing agent, induces p53-dependent and p53-independent glioma cell death. Oncogene. 2003;22(51):8233–45. doi:10.1038/sj.onc.1207198 Cited in: PubMed; PMID 14614447.

5. Eckerich C, Schulte A, Martens T, et al. RON receptor tyrosine kinase in human gliomas: expression, function, and identification of a novel soluble splice variant. J Neurochem. 2009;109(4):969–80. doi:10.1111/j.1471-4159.2009.06027.x Cited in: PubMed; PMID 19519771.

6. Bruns I, Sauer B, Burger MC, et al. Disruption of peroxisome proliferator-activated receptor γ coactivator (PGC)-1α reverts key features of the neoplastic phenotype of glioma cells. J Biol Chem. 2019;294(9):3037–50. doi:10.1074/jbc.RA118.006993 Cited in: PubMed; PMID 30578297.

7. Golebiewska A, Hau A-C, Oudin A, et al. Patient-derived organoids and orthotopic xenografts of primary and recurrent gliomas represent relevant patient avatars for precision oncology. Acta Neuropathol. 2020. doi:10.1007/s00401-020-02226-7 Cited in: PubMed; PMID 33009951.

8. McAlister GC, Nusinow DP, Jedrychowski MP, et al. MultiNotch MS3 enables accurate, sensitive, and multiplexed detection of differential expression across cancer cell line proteomes. Anal Chem. 2014;86(14):7150–8. doi:10.1021/ac502040v Cited in: PubMed; PMID 24927332.

9. R Core TeamR Core TeamR Core TeamR Core Team. R: A language and environment for statistical computing. R Foundation for Statistical Computing, Vienna, Austria. [Internet]. 2022. Available from: https://www.R-project.org/.

10. RStudio TeamRStudio TeamRStudio TeamRStudio Team. RStudio: Integrated Development for R. RStudio, PBC, Boston, MA [Internet]. 2020. Available from: http://www.rstudio.com/

**Supplementary Tables**

**Supplementary Table 1**

| Gene | Function |
| --- | --- |
| KPNB1 | Involved in nuclear import of proteins, including transcription factors that regulate mitochondrial gene expression. |
| HMGB1 | Plays a role in mitochondrial DNA replication and transcription, as well as regulation of oxidative stress and apoptosis. |
| TPM1 | May be involved in the organization and stabilization of the mitochondrial network. |
| HMGB2 | Involved in mitochondrial DNA maintenance and oxidative stress response. |
| U2AF2 | Participates in the splicing of mitochondrial pre-mRNAs, affecting gene expression and mitochondrial function. |
| TOMM40 | A component of the translocase of the outer mitochondrial membrane (TOM complex) involved in protein import into mitochondria. |
| AK2 | Encodes adenylate kinase 2, which is important for maintaining cellular energy homeostasis, including mitochondrial ATP production. |
| NANS | Enzyme involved in the biosynthesis of sialic acid, which has implications for mitochondrial metabolism and oxidative stress. |
| DFFA | Involved in apoptosis, but also plays a role in mitochondrial fragmentation and clearance. |
| CPSF6 | Contributes to mitochondrial RNA processing and stability. |
| CSNK2B | Encodes the regulatory subunit of casein kinase 2, which has been associated with mitochondrial function and apoptosis. |
| UTP14A | Likely involved in mitochondrial ribosome assembly and translation of mitochondrial-encoded proteins. |
| NOLC1 | Participates in the regulation of mitochondrial protein synthesis. |

Supplementary Table 1. Summary of genes related to mitochondrial metabolism that are linked to overexpression of PGC-1α based on the WGCNA-analysis. Gene name and the proposed function are annotated.

**Supplementary Table 2**

| Gene | Forward | Reverse |
| --- | --- | --- |
| 18s | 5-CGGCTACCACATCCAAGGAA-3′ | 5-GCTGGAATTACCGCGGCT-3′ |
| SDHA | 5-TGGGAACAAGAGGGCATCTG-3′ | 5-CCACCACTGCATCAAATTCATG-3′ |
| PGC-1α | 5-TCTGAGTCTGTATGGAGTGACAT3′ | 5-CCAAGTCGTTCACATCTAGTTCA-3′ |
| GALT | 5-CGCAGTGGAACCGATCCTC-3′ | 5-GATGGTCGTTTGCCCGGAA-3′ |
| GALE | 5-CTGGAGGCTGGCTACTTGC-3′ | 5-CCCTGGTCCAAAATGTCCATCT-3′ |
| OXCT1 | 5-CACCAGTGCTCATCGCCATA-3′ | 5-CACATAGCCCAAAACCACCAA-3′ |
| ACAT1 | 5-GGAGAGCATGTCCAATGTTCC-3′ | 5-CGTCCTGTTCATTTCGTGCAA-3′ |
| CPT1c | 5-ATGGGAATGCGCCCCTTATG-3 | 5-AGGTGGCGGATGTAGTCTTTT-3 |
| HADH | 5-ATATGCCGCAATTTTACAGGGT-3 | 5-ACCTGCAATAAAGCAGCAGCCTGG-3 |
| ATP5g1 | 5-CTGTTGTACCAGGGGTCTAATCA-3′ | 5-GTGGGAAGTTGCTGTAGGAAG-3′ |
| MT-D-Loop | 5-GTGAAATCAATATCCCGCACAAGAG-3′ | 5-TATGACCCTGAAGTAGGAACCAGA-3′ |
| MT-CO1 | 5-CCCTAGACCAAACCTACGCC-3′ | 5-ATGTGGTGTATGCATCGGGG-3′ |
| MT-CO2 | 5- CCGTCTGAACTATCCTGCCC-3′ | 5- AAGCCTAATGTGGGGACAGC-3′ |
| MT-CYB | 5-ACCCCCTAGGAATCACCTCC-3′ | 5-GCCTAGGAGGTCTGGTGAGA-3′ |
| NRF1 | 5-AGGAACACGGAGTGACCCAA-3′ | 5-TGCATGTGCTTCTATGGTAGC-3′ |
| SOD1 | 5-GGTGGGCCAAAGGATGAAGAG-3′ | 5-CCACAAGCCAAACGACTTCC-3′ |
| SOD2 | 5- TGGACAAACCTCAGCCCTAA-3′ | 5- GCGTTGATGTGAGGTTCCAG-3′ |

Supplementary Table 2.

List of primer pairs used for qRT-PCR analysis

**Supplementary Table 3**

| Antibody | Dilution factor | Company | Cat. Nr. |
| --- | --- | --- | --- |
| PGC-1α | 1:1000 | Cell Signaling Technology  (Danvers, MA, USA) | #2178 |
| P38 MAPK | 1:1000 | Cell Signaling Technology | #9212 |
| Phospho-p38 MAPK (Thr180/Tyr182) | 1:750 | Cell Signaling Technology | #9211 |
| AMPKα1/2 | 1:1000 | Cell Signaling Technology | #2532 |
| AMPKα2 | 1:1000 | Cell Signaling Technology | #2757 |
| Phospho-AMPKα (Thr172) | 1:1000 | Cell Signaling Technology | #2535 |
| Acetyl CoA carboxylase | 1:500 | Cell Signaling Technology | #3662 |
| Phospho-Acetyl-CoA Carboxylase, | 1:500 | Cell Signaling Technology | #3661 |
| AMPKα1 | 1:1000 | Abcam (Cambridge, UK) | #ab32047 |
| Actin | 1:2000 | Santa Cruz Biotechnology  (Santa Cruz, CA, USA). | #sc-1616 |
| Anti-mouse secondary | 1:3000 | Santa Cruz Biotechnology | #sc-516102 |
| Anti-goat secondary | 1:5000 | Santa Cruz Biotechnology | #sc-2020 |
| Anti-rabbit-secondary | 1:8000 | Jackson ImmunoResearch  (West Grove, PA, USA) | #111-036-144 |

Supplementary Table 3. List of primary and secondary antibodies used in this article
